# Efficacy and safety of duloxetine for osteoarthritis: a comparator-stratified systematic review and meta-analysis

**DOI:** 10.1016/j.eclinm.2026.104296

**Published:** 2026-10-29

**Authors:** Di Zhang, Arashi Shahid, Gege Zhang, Bo Xin, Leticia A. Deveza, Venkatesha Venkatesha, David J. Hunter

**Affiliations:** a School of Public Health, Xi'an Jiaotong University Health Science Center, Xi'an, PR China; b Sydney Musculoskeletal Health, Kolling Institute of Medical Research, The University of Sydney, NSW, Australia; c National and Local Joint Engineering Research Center of Biodiagnosis and Biotherapy, The Second Affiliated Hospital of Xi'an Jiaotong University, Xi'an, Shaanxi, PR China; d Yan'an Medical College, Yan'an University, Yan'an, PR China; e School of Nursing, Xi'an Jiaotong University, Xi'an, PR China; f Rheumatology Department, Royal North Shore Hospital, St Leonards, Australia; g Faculty of Eastern Medicine, Hamdard University, Karachi, Pakistan

**Keywords:** Duloxetine, Osteoarthritis, Pain, Comparator-stratified meta-analysis, Physical function, Randomised controlled trials

## Abstract

**Background:**

Duloxetine is recommended for osteoarthritis pain, yet its role relative to placebo, active pharmacological comparators, and add-on therapy remains uncertain. We assessed the efficacy and safety of duloxetine for osteoarthritis using a comparator-stratified meta-analysis.

**Methods:**

We searched major English and Chinese databases from inception to August 30, 2026, for randomised controlled trials of duloxetine in adults with osteoarthritis. Eligible trials compared duloxetine with placebo, active pharmacological treatments, or add-on/background therapy controls. Pain and physical function were the primary outcomes. We used random-effects meta-analysis, comparator-stratified and duration-based subgroup analyses, meta-regression, sensitivity analyses, and Grading of Recommendations Assessment, Development and Evaluation (GRADE) certainty assessment.

**Findings:**

20 studies, contributing 21 eligible randomised comparisons and 3455 participants, were included. Duloxetine was associated with reduced Western Ontario and McMaster Universities Osteoarthritis Index (WOMAC) pain (standardised mean difference −0.36, 95% CI −0.58 to −0.14) and improved physical function (−0.41, −0.58 to −0.25), but with more adverse events overall (risk ratio 1.36, 95% CI 1.03–1.79). Comparator-stratified analyses were conducted separately for placebo-controlled, active-comparator, and add-on/background-therapy settings, which addressed distinct clinical questions. Placebo-controlled studies provided the clearest evidence of benefit, whereas duloxetine did not show clear superiority over active pharmacological comparators. Add-on or combination-therapy estimates for pain and function were inconclusive: the confidence intervals crossed the null and the certainty of evidence was very low. No statistically significant associations were detected between maintenance dose, treatment duration, or study quality and WOMAC treatment effects; however, these exploratory analyses were based on a limited number of studies. Certainty of evidence ranged from low to high; evidence was low for WOMAC pain and total scores, and overall adverse events, and moderate for WOMAC physical function, stiffness.

**Interpretation:**

Duloxetine was associated with improvements in pain and physical function in osteoarthritis, with the clearest benefit observed versus placebo. Evidence from active-comparator and add-on/background-therapy studies was limited, heterogeneous, and inconclusive; these analyses did not establish superiority over active treatment or a consistent additive treatment effect. Duloxetine may be considered a conditional pharmacological option for osteoarthritis pain management, particularly when non-steroidal anti-inflammatory drugs are unsuitable; however, its comparative benefit over active treatments remains uncertain.

**Funding:**

Shaanxi Postdoctoral Research Program, Natural Science Basic Research Program of Shaanxi, Fundamental Research Funds for the Central Universities, China Postdoctoral Science Foundation, and NHMRC Investigator Grant.

**Research in context**

**Evidence before this study**

We searched major English and Chinese biomedical databases, clinical trial registries, and reference lists of relevant reviews for systematic reviews, meta-analyses, and randomised controlled trials evaluating duloxetine in osteoarthritis, from database inception to August 30, 2026. Previous reviews generally reported that duloxetine improved pain and physical function in osteoarthritis. However, most previous reviews pooled placebo-controlled trials, active-comparator trials, and add-on or combination-therapy studies together, limiting the ability to determine whether duloxetine provides a clinically meaningful benefit beyond established pharmacological treatments or when used as part of combination therapy. Evidence regarding psychological outcomes, quality of life, adverse-event profiles, and tolerability was also incompletely or inconsistently reported, and several recently published randomised controlled trials had not been included in earlier reviews.

**Added value of this study**

To our knowledge, this is the first comparator-stratified meta-analysis evaluating duloxetine across placebo-controlled, active-comparator, and add-on or combination-therapy settings in osteoarthritis. By synthesising evidence from 20 studies contributing 21 eligible randomised comparisons and involving 3455 participants, we found that duloxetine was associated with modest improvements in pain and physical function overall, with the clearest and most consistent evidence observed in placebo-controlled comparisons. In contrast, duloxetine did not clearly outperform active pharmacological comparators. Evidence from add-on or combination-therapy studies was limited and inconclusive because of heterogeneous background treatments, small numbers of studies, and imprecise estimates. We also assessed psychological outcomes, adverse events, treatment duration, and potential sources of heterogeneity using subgroup analyses, meta-regression, sensitivity analyses, and Grading of Recommendations Assessment, Development and Evaluation (GRADE) certainty assessment.

**Implications of all the available evidence**

The available evidence suggests that duloxetine should not be viewed as a broadly superior alternative to existing pharmacological therapies for osteoarthritis. Rather, its role is best considered as a conditional pharmacological option within multimodal osteoarthritis pain management, particularly when non-steroidal anti-inflammatory drugs (NSAIDs) are unsuitable or poorly tolerated. Future trials should evaluate prespecified clinical characteristics as potential effect modifiers, evaluating long-term effectiveness and discontinuation strategies, directly comparing duloxetine with active analgesic options, and consistently reporting harms and tolerability, while also clarifying the effects of duloxetine on psychological outcomes and quality of life.

## Introduction

Osteoarthritis is a highly prevalent chronic joint disease and a major contributor to disability worldwide.^1^ It is characterised by progressive cartilage degradation, subchondral bone remodelling, chronic pain, and functional impairment, which substantially reduce patients’ quality of life.^2^ With the ageing global population and increasing prevalence of obesity, the burden of osteoarthritis is expected to rise further, posing a significant individual and societal burden on individuals, health systems, and societies.^3^

Osteoarthritis management combines non-pharmacological approaches, including exercise and weight management, with pharmacological therapies such as acetaminophen, non-steroidal anti-inflammatory drugs (NSAIDs), topical analgesics, and intra-articular injections.^3^, ^4^ However, these treatments often provide limited and temporary relief, and many patients continue to experience chronic pain and reduced quality of life. Psychological comorbidities are common in osteoarthritis patients, with approximately 40% of individuals with osteoarthritis also suffering from anxiety or depression, which further exacerbates disease burden and complicates management.^5^, ^6^ Duloxetine, a serotonin–norepinephrine reuptake inhibitor, is used for major depressive disorder, generalised anxiety disorder, neuropathic pain, chronic musculoskeletal pain, and fibromyalgia.^7^ Its analgesic effect is believed to involve modulation of descending inhibitory pain pathways in the central nervous system. Furthermore, by simultaneously addressing mood disturbances and pain, duloxetine may provide dual benefits for patients with osteoarthritis, who often suffer from both physical and psychological symptoms.^8^

Several previous randomised controlled trials (RCTs) have shown that duloxetine may relieve pain and improve physical function in patients with osteoarthritis.^9^, ^10^, ^11^, ^12^ Previous meta-analyses have also evaluated duloxetine in osteoarthritis and generally reported beneficial effects on pain and function.^13^, ^14^, ^15^, ^16^, ^17^ Broader reviews of antidepressants for musculoskeletal pain and osteoarthritis have suggested that serotonin–noradrenaline reuptake inhibitors, including duloxetine, provide small improvements in pain and disability in osteoarthritis, but are associated with more adverse events.^18^ Evidence from a chronic pain network meta-analysis also suggests that duloxetine has among the most certain evidence within the antidepressant class, although evidence on long-term outcomes, safety, and patient subgroups remains limited.^19^ A Cochrane review focused on antidepressants for hip and knee osteoarthritis found small benefits for pain and function, but the available osteoarthritis evidence was largely limited to placebo-controlled trials.^8^ However, most reviews pooled studies with different comparator types together, including placebo, active pharmacological treatments, and combination therapies, which may obscure important differences in treatment effects across clinical settings. Consequently, it remains unclear whether the observed benefits of duloxetine are driven primarily by placebo-controlled studies or whether duloxetine provides additional advantages over established pharmacological treatments. In addition, the effects of duloxetine on depression and anxiety outcomes in osteoarthritis remain uncertain, and several recent trials have not been included in earlier reviews.

To address these knowledge gaps, we conducted a systematic review and comparator-stratified meta-analysis of RCTs evaluating duloxetine in osteoarthritis. We aimed to synthesise the evidence separately according to comparator category, because these settings address distinct clinical questions. We also aimed to assess its efficacy and safety within each comparator category and to explore potential sources of heterogeneity through subgroup, sensitivity, and meta-regression analyses, thereby clarifying where duloxetine may be positioned within individualised osteoarthritis pain management.

## Methods

### Search strategy and selection criteria

A systematic search was conducted in five English-language and three Chinese biomedical databases from inception to Aug 30, 2026. The databases searched were PubMed, Embase, Web of Science, Scopus, and the Cochrane Central Register of Controlled Trials (CENTRAL), and the China National Knowledge Infrastructure (CNKI), Chongqing VIP Information (CQVIP), and Wanfang Data. In addition, ClinicalTrials.gov and the reference lists of previously published reviews on duloxetine and osteoarthritis were screened to identify eligible clinical trials. To minimise the risk of missing relevant studies, a manual search of related systematic reviews was also performed. The search strategy combined Medical Subject Headings (MeSH) and free-text terms, including but not limited to: “osteoarthritis”, “knee osteoarthritis”, “hip osteoarthritis”, “hand osteoarthritis”, “duloxetine”, “duloxetine hydrochloride” and “randomised controlled trials (RCTs)”. No restrictions were placed on the language of publication. For specific search strategies, see Supplement_Merh.

Participants in eligible studies were adults diagnosed with osteoarthritis according to established clinical and radiological criteria (e.g., the American College of Rheumatology). Studies of knee, hip, or hand osteoarthritis were eligible when osteoarthritis-specific data were extractable. Trials enrolling mixed arthritis populations were excluded unless data specific to osteoarthritis participants could be extracted. All participants were aged 18 years or older. Randomised controlled trials evaluating duloxetine either as monotherapy or as add-on therapy were eligible. Comparators could include placebo, active pharmacological treatment, usual care, or background therapy, with a minimum intervention duration of 2 weeks. Because placebo-controlled trials, active-comparator trials, and trials evaluating duloxetine as add-on or combination therapy address different clinical treatment questions, these treatment settings were analysed separately according to comparator type.

Studies involving mixed populations were considered eligible if data specific to individuals with knee, hip, or hand osteoarthritis could be extracted. Trials enrolling participants with other types of arthritis (e.g., rheumatoid arthritis) were excluded. In addition, studies were excluded if they had no control group, included fewer than 20 participants, or were case reports/series, review articles, conference abstracts, commentaries, editorials, animal studies, cadaveric studies, or other designs without extractable clinical data.

The review protocol was prospectively registered in the International Prospective Register of Systematic Reviews, CRD42023481338. This systematic review and meta-analysis was reported in accordance with the PRISMA 2020 statement.

### Ethics

Ethical approval was not required because this systematic review and meta-analysis used data from published studies and did not involve individual participant data.

### Data extraction and outcomes

Search results were imported into EndNote and then into Covidence for study management. Reviewers (DZ, AS, GGZ and BX) independently screened titles/abstracts and full texts and removed duplicates. Disagreements were resolved by a third reviewer (DJH), and reasons for exclusion were recorded. Trial authors were contacted for clarification when necessary. Data were extracted using piloted forms in Covidence, including study characteristics (author, year, journal, design), participant details (sample size, age, sex, BMI, osteoarthritis site, disease duration), setting (country, health care context), intervention and comparator characteristics, treatment duration, and outcome data on pain, physical function, stiffness, health-related quality of life, global assessments, psychological symptoms, treatment failure or ineffective response, and safety.

The use of a specific outcome instrument was not an eligibility requirement. For each outcome domain, data were extracted from validated measures reported by eligible studies. The primary outcomes were pain and physical function. Pain outcomes comprised pain intensity or severity assessed using validated measures, including, but not limited to, the Western Ontario and McMaster Universities Osteoarthritis Index (WOMAC) pain subscale, Brief Pain Inventory-Severity (BPI–S), visual analogue scale (VAS), numerical rating scale (NRS), 24-h average or worst pain ratings, and the Australian/Canadian Hand Osteoarthritis Index (AUSCAN) pain subscale. Pain responder outcomes, where reported, were defined as the proportions of participants achieving at least 30% or 50% reduction in pain. Physical function was assessed using validated measures such as the WOMAC physical-function and AUSCAN function subscales. Pain-related functional interference was assessed separately from physical function using the Brief Pain Inventory-Interference (BPI–I), and stiffness was analysed as a separate symptom outcome where reported.

Secondary outcomes included health-related quality of life, global assessments, psychological symptoms, treatment failure or ineffective response, and safety outcomes. Health-related quality of life was assessed using measures such as the 36-Item Short-Form Health Survey (SF-36) and EQ-5D. Global assessments included patient- or clinician-reported measures such as the Patient Global Impression of Improvement (PGI-I) and Clinical Global Impression measures. Psychological symptoms were classified as depression or anxiety according to the construct assessed by the original instrument. Depression was assessed using validated depression measures such as the Beck Depression Inventory-II (BDI-II), the Geriatric Depression Scale, and the depression subscale of the Hospital Anxiety and Depression Scale (HADS-D), where reported. Anxiety was assessed using validated anxiety measures such as the HADS anxiety subscale (HADS-A) and the Hamilton Anxiety Rating Scale (HAMA). Broader mood-related or mental-health-related domains, such as SF-36 mental health and BPI-I mood, were analysed under their parent instruments and were not pooled with depression/anxiety-specific scales because they measure different constructs. Anxiety analyses were considered exploratory secondary analyses. Safety outcomes included overall adverse events, serious adverse events, and treatment discontinuation due to adverse events, as reported by the original trial investigators. These outcomes were treated as distinct safety endpoints. Trial-defined treatment failure or ineffective response was analysed as a binary outcome when reported.

For the primary analysis, outcome data were extracted at the end of the randomised treatment period. When multiple assessments were reported during treatment, the assessment closest to treatment completion was selected to provide one comparable effect estimate per study and outcome and to avoid repeated inclusion of correlated measurements from the same participants. Post-treatment follow-up assessments were not included in the primary meta-analysis because they reflected persistence of treatment effects after completion of therapy rather than efficacy during active treatment, and follow-up schedules were not consistently reported across studies. For continuous secondary outcomes, data were extracted at the selected treatment endpoint where available, while adverse-event data were extracted over the randomised treatment period. To provide a transparent link between the extracted data and the pooled analyses, we created a study-to-analysis mapping table listing, for each included study, the eligible comparison(s), comparator category, reported outcome measure(s), time point, corresponding meta-analysis, and multi-arm handling where applicable (Supplementary Table S4).

### Risk of bias assessment

The risk of bias for each included study was assessed using the Cochrane Risk of Bias tool version 1 (RoB 1) for Randomised Trials,^20^ which considers the following domains: selection bias (random sequence generation and allocation concealment), blinding of participants, personnel and outcome assessment, incomplete outcome data, selective reporting, and other potential sources of bias. Each domain was judged as having a low, high, or unclear risk of bias in accordance with the criteria specified in the Cochrane RoB 1 guidance. A low-risk judgement indicated that the reported methods were adequate to minimise bias in that domain; a high-risk judgement indicated methodological features likely to introduce bias; and an unclear-risk judgement was assigned when the information reported was insufficient to permit a definitive assessment. Two reviewers independently assessed risk of bias, and any disagreements were resolved through discussion with a third reviewer.

### Certainty of evidence assessment

The certainty of evidence for key outcomes was assessed using the Grading of Recommendations Assessment, Development and Evaluation (GRADE) framework.^21^ Evidence from RCTs was initially rated as high certainty and was downgraded when concerns were identified regarding risk of bias, inconsistency, indirectness, imprecision, or publication bias. The certainty of evidence for each key outcome was categorised as high, moderate, low, or very low. Two reviewers independently performed the GRADE assessments for each outcome. Disagreements were resolved through discussion and consensus, with consultation of a third reviewer when necessary.

### Statistics

For continuous outcomes, standardised mean differences (SMDs) were calculated as Cohen's d by dividing the difference in mean outcome between the duloxetine and comparator groups by the pooled within-study standard deviation, with corresponding 95% CIs. Effect directions were harmonised across studies so that the direction of benefit was consistent within each outcome. Binary outcomes were analysed using risk ratios (RRs) with corresponding 95% CIs. The main safety meta-analysis assessed overall adverse events. Serious adverse events and treatment discontinuation due to adverse events were analysed separately where data were available. In accordance with Cohen's conventional criteria, SMDs of approximately 0.2, 0.5, and 0.8 were interpreted as small, moderate, and large effect sizes, respectively. These thresholds were used to describe the magnitude of standardised effects and were not interpreted as established minimal clinically important differences. Between-study heterogeneity was assessed using Cochran's Q statistic and quantified using the I^2^ statistic, with I^2^ of less than 50% indicating low heterogeneity and I^2^ of 50% or more considered substantial heterogeneity. Meta-analyses were performed using random-effects models with restricted maximum likelihood estimation of the between-study variance. Given the small number of studies contributing to several analyses, Hartung-Knapp-Sidik-Jonkman adjustment was applied to confidence intervals and hypothesis tests. Given the clinical differences between comparator settings, comparator-stratified analyses were used as the primary basis for clinical interpretation. Comparator categories were analysed separately to describe the evidence according to the clinical question addressed. No formal between-category comparison or interaction test was undertaken; therefore, differences in subgroup-specific pooled estimates were not interpreted as evidence that treatment effects differed across comparator categories. Overall pooled estimates, where presented, were considered secondary descriptive summaries and were not interpreted as representing a single common treatment effect across comparator settings. Additional subgroup analyses according to osteoarthritis site and severity, baseline pain intensity, symptom duration, pain phenotype, duloxetine dosing and titration, and concomitant or background therapy were not performed because these characteristics were inconsistently reported and the number of studies within individual categories was limited. Outcomes and subscales were meta-analysed separately. When multiple outcomes or domains were reported for the same participants, these effect estimates were not combined into a single cross-outcome pooled estimate because of within-study correlation. For multi-arm trials, when more than one comparison contributed to the same meta-analysis, shared intervention or comparator groups were split to avoid double counting participants. For continuous outcomes, we divided the shared-group sample size across comparisons while retaining means and standard deviations. For binary outcomes, shared event counts and total sample sizes were divided across comparisons. When only one eligible comparison from a multi-arm trial contributed to a given meta-analysis, no splitting was required.

Sensitivity analyses were conducted when substantial heterogeneity was observed. Leave-one-out sensitivity analyses were performed by sequentially omitting each study from the meta-analysis and recalculating the pooled effect estimate and heterogeneity statistics after each omission. These analyses were used to assess whether the pooled estimate or between-study heterogeneity was disproportionately influenced by any single study. Because directional stability after study exclusion does not necessarily indicate clinical homogeneity or certainty in the magnitude of the pooled effect, the leave-one-out analyses were interpreted as assessments of individual-study influence rather than as definitive evidence of robustness. To further explore potential sources of heterogeneity, exploratory random-effects meta-regression analyses were performed using maintenance dose (mg/day), treatment duration (weeks), and study quality score as study-level covariates. Meta-regression analyses were conducted for the primary WOMAC outcomes (total score, pain, physical function, and stiffness), and the proportion of between-study heterogeneity explained by each covariate was quantified using the R^2^ statistic. Pain and function outcomes were analysed separately by instrument or domain rather than combined across measures. This approach was used because the instruments differed in construct, recall period, scaling, and clinical interpretation, and because several studies reported multiple correlated measures from the same participants. Additionally, treatment-duration subgroup analyses were based on the duration of the randomised treatment period (<12 weeks versus ≥12 weeks), rather than the duration of post-treatment follow-up. Differences between subgroups were evaluated using tests for subgroup interaction. Publication bias was assessed visually using funnel plots and statistically using Egger's regression test. All statistical tests were two-sided, and a p value of less than 0.05 was considered statistically significant. Statistical analyses were conducted using STATA version 18.0.

### Role of the funding source

The funders had no role in study design, data collection, data analysis, data interpretation, writing of the report, or the decision to submit for publication.

## Results

The database search identified 2074 records. After removing 1080 duplicates, 994 records were screened. Following title and abstract screening, 58 full-text articles were assessed for eligibility, of which 38 were excluded for various reasons. Finally, 20 studies were included in the systematic review and meta-analysis. One multi-arm trial contributed two eligible randomised comparisons, giving 21 eligible comparisons in total. Shared groups were split when multiple comparisons from the same trial entered the same pooled analysis (Fig. 1a).

**Fig. 1:**
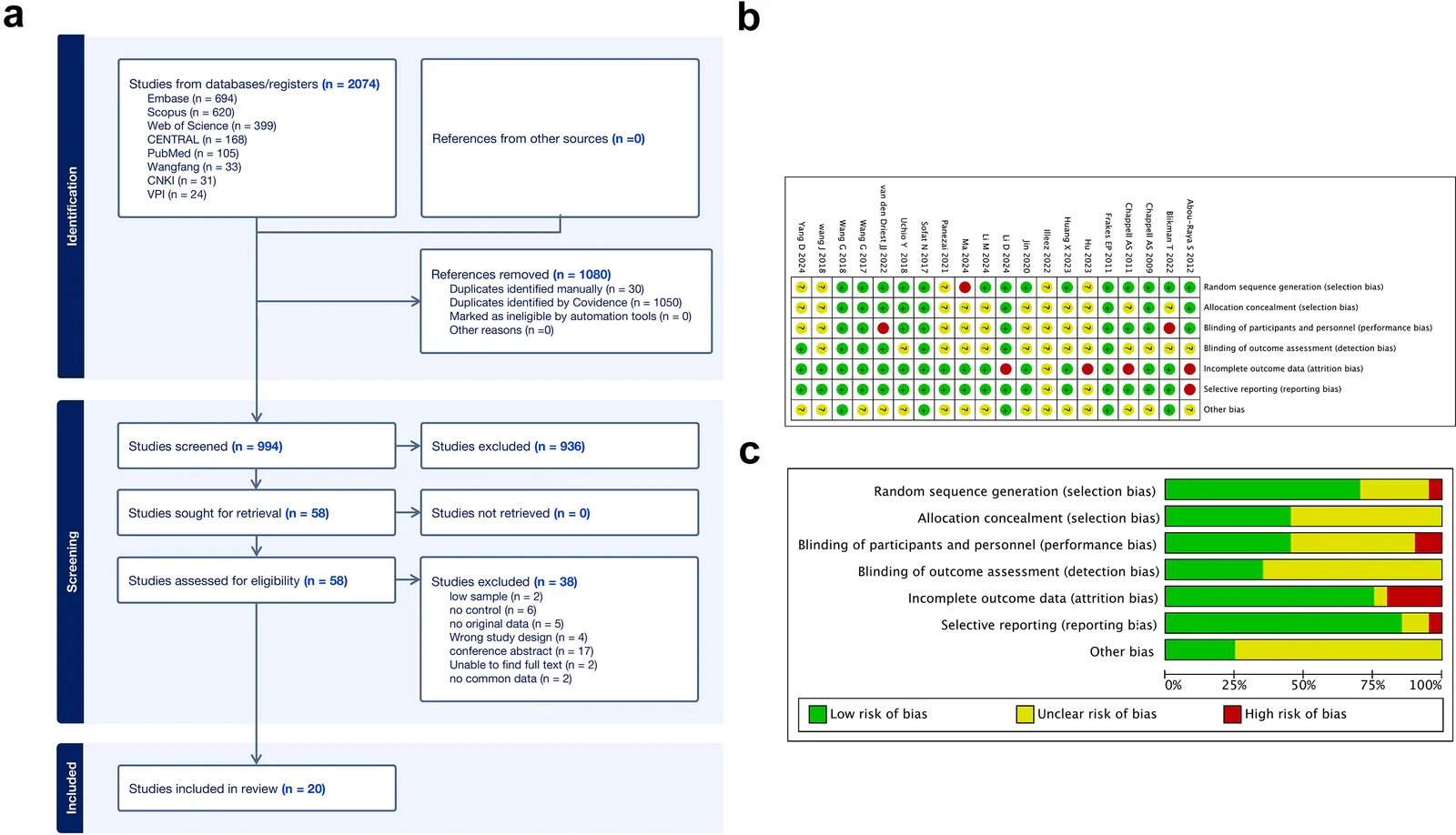
**Study selection and risk-of-bias assessment.** (a) PRISMA flow diagram showing the identification, screening, eligibility assessment, and inclusion of studies. (b) Risk-of-bias assessment for each included randomised controlled trial using the Cochrane Risk of Bias tool. (c) Summary of risk-of-bias judgements across all included studies. Abbreviation: PRISMA, Preferred Reporting Items for Systematic Reviews and Meta-Analyses.

20 RCTs, contributing 21 eligible randomised comparisons, were included, with sample sizes per arm ranging from 21 to 264 participants. Studies were conducted in the United States (n = 3),^22^, ^23^, ^24^ China (n = 10),^9^, ^12^, ^25^, ^26^, ^27^, ^28^, ^29^, ^30^, ^31^, ^32^ Japan (n = 1),^33^ the Netherlands (n = 2),^11^, ^34^ the United Kingdom (n = 2),^35^ Egypt (n = 1),^36^ Pakistan (n = 1),^37^ and Turkey (n = 1).^38^ Participants were mainly women, aged 48.9–74.4 years, with a mean BMI of 22.6–31.7 kg/m^2^ and osteoarthritis symptom duration of 1.4–9.8 years. Most trials involved knee osteoarthritis (n = 16); three included both knee and hip osteoarthritis, two investigated hand osteoarthritis, and the osteoarthritis site was not reported in two studies. Comparator interventions varied across the included studies and were classified according to the clinical question addressed. Of the 21 eligible randomised comparisons, seven were placebo-controlled, seven used active pharmacological or usual-care comparators, and seven evaluated duloxetine as add-on or combination therapy. Active pharmacological or usual-care comparators included NSAIDs, celecoxib, meloxicam, pregabalin, and usual care. Add-on or combination-therapy studies evaluated duloxetine added to background treatment, including NSAIDs, loxoprofen plus glucosamine, radiofrequency therapy, surgery, usual care, or intra-articular hyaluronic acid plus corticosteroid injection. Because these comparator types address different clinical questions, the analyses were stratified by comparator category. Treatment duration ranged from 7 weeks to 24 months. Although the eligibility criteria permitted a minimum intervention duration of 2 weeks, the shortest treatment duration among included studies was 7 weeks. Outcomes assessed included pain, physical function, stiffness, health-related quality of life, psychological symptoms, global assessments, and safety outcomes. Baseline characteristics are summarised in Table 1.

**Table 1:** Characteristics of included studies and treatment interventions.

Number	Author	Journal	DOI	Country	NCT	Number (Men/Women)		Age		Duration of OA pain, years mean (SD)		BMI		Group		Time (Week)	Site	Outcomes
						Intervention	Control	Intervention	Control	Intervention	Control	Intervention	Control	Intervention	Control			
1	Chappell 2011^22^	Pain Practice	10.1111/j.1533-2500.2010.00401.x	USA	NCT00433290	128 (39/89)	128 (21/107)	63.2 (8.8)	61.9 (9.2)	6.2 (5.9)	5.6 (6.2)	/	/	Duloxetine	Placebo	13 W	knee	BPI-S ratings BPI-I ratings BPI average pain 24-h average pain score 24-h worst pain score CGI-Severity WOMAC SF-36 PGI AEs
2	Uchio 2018^33^	J Pain Res	10.2147/JPR.S164128	Japan	NCT02248480	177 (35/142)	176 (44/132)	65.5 (8.0)	66.4 (8.4)	4.0 (4.2)	4.5 (4.3)	25.8 (4.4)	25.7 (3.9)	Duloxetine	Placebo	14 W	knee	BPI-S ratings BPI-I ratings 24-h average pain score 24-h worst pain score BPI average pain WOMAC CGI-Severity SF-36 PGI EQ-5D-5L score SAEs
3	Sofat 2017^35^	Journal of Pain Research	10.2147/JPR.S147640	UK	NCT02612233	21 (7/14)	22 (3/19)	62.3 (7.3)	62.4 (8.7)	3.5 (4.2)	3.5 (4.2)	28.4 (5.9)	27.0 (4.3)	Duloxetine	Placebo	13 W	Hand	NRS AUSCAN function AUSCAN pain score AUSCAN stiffness SAEs HADS-D (Depression) HADS-A (Anxiety)
							22 (3/19)		64.0 (5.0)		3.5 (4.2)		27.1 (6.3)	Duloxetine	Pregabalin			
4	Frakes 2011^24^	Current Medical Research & Opinion	10.1185/03007995.2011.633502	USA	NCT01018680	264 (112/152)	260 (113/147)	61.9 (9.2)	60.3 (9.2)	9.8 (8.9)	9.2 (8.9)	31.7 (5.6)	31.5 (5.5)	Duloxetine	Placebo	8 W	knee	BPI-S ratings BPI-I ratings WOMAC PGI SAEs
5	Chappell 2009^23^	Pain	10.1016/j.pain.2009.06.024	USA	/	111 (41/70)	120 (39/81)	62.1 (9.6)	62.5 (9.3)	9.0 (8.7)	9.3 (8.3)	30.69 (6.65)	31.59 (6.65)	Duloxetine	Placebo	13 W	knee	BPI-S ratings BPI-I ratings 24-h average pain score 24-h worst pain score BPI average pain CGI-Severity WOMAC SF-36 PGI EQ-5D-5L score HADS-A (Anxiety) AEs
6	Abou-Raya 2012^36^	Age and Ageing	10.1093/ageing/afs072	Egypt	NCT01425827	144 (23/121)	144 (24/120)	68.9 (6.2)	68.5 (5.8)	5.7 (4.9)	5.6 (4.5)	27.6 (4.6)	27.5 (5.1)	Duloxetine	Placebo	16 W	knee	WOMAC HAMA
7	Wang G 2017^12^	Osteoarthritis and Cartilage	10.1016/j.joca.2016.12.025	China	NCT01931475	205 (45/160)	202 (51/151)	61.2 (8.2)	59.8 (8.4)	8.2 (7.8)	7.8 (7.1)	25.4 (3.2)	25.4 (3.7)	Duloxetine	Placebo	13 W	Hip/Knee	BPI-S ratings BPI-I ratings BPI average pain CGI-Severity WOMAC PGI AEs
8	Wang 2018^27^	People's Armed Police Medical Corps	10.14010/j.cnki.wjyx.2018.07.020	China	/	63	57	30–70		>3 M		/	/	Duloxetine Hydrochloride Enteric-Coated + Loxoprofen Sodium + Glucosamine Sulphate	Loxoprofen Sodium + Glucosamine Sulphate	8 W	knee	NRS HAMD SAEs
9	Huang 2023^31^	Chinese Journal of Pain Medicine	10.3760/cma.j.cn101658-20210407-00048	China	/	24 (4/20)	24 (5/19)	67 (11)	65 (12)	27 (34)	33 (39)	22.8 (1.8)	22.6 (2.2)	Duloxetine + Radiofrequency therapy	Radiofrequency therapy	8 W	knee	VAS WOMAC
10	Jin 2020^30^	Zhejiang Practical Medicine	10.16794/j.cnki.cn33-1207/r.2020.03.012	China	/	36 (12/24)	36 (13/23)	61.2 (8.7)	60.6 (8.2)	/	/	/	/	Duloxetine_+_ Surgery	Surgery	8 W	knee	VAS
11	Ma 2024^32^	Chinese Medical Science Journal Database	/	China	/	29 (11/18)	28 (12/16)	74.19 (2.96)	74.38 (2.85)	/	/	23.45 (1.28)	23.17 (1.13)	Duloxetine	Celecoxib		knee	VAS WOMAC
12	Yang 2024^26^	Chinese Science and Technology Journal Database	/	China	/	32 (15/17)	33 (16/17)	60.69 (9.27)	61.29 (8.84)	3.86 (1.45)	3.68 (1.26)	/	/	Duloxetine	Celecoxib + Ibuprofen	8 W	knee	VAS
13	van den Driest 2022^34^	Arthritis & Rheumatology	10.1002/art.42040	Netherlands	NTR4798	66 (27/39)	66 (16/50)	63.2 (10.5)	65.4 (11.2)	7.8 (6.5)	9.2 (8.2)	30.6 (6.6)	30.9 (6.2)	Duloxetine + usual care	Usual care		Hip/Knee	WOMAC NRS HADS-D (Depression) HADS-A (Anxiety)
14	Blikman 2022^11^	BMC Musculoskeletal Disorder	10.1186/s12891-022-05034-0	Netherlands	NTR4744	57 (19/38)	54 (23/31)	61.5 (8.1)	64 (8.7)	4.0 (4.17)	3.0 (3.6)	28.8 (4.9)	29 (3.9)	Duloxetine	Usual care	7 W	Hip/Knee	VAS PGI
15	Panezai 2021^37^	P J M H S	10.53350/pjmhs2115102865	Pakistan	/	64 (16/48)	64 (22/42)	48.94 (5.8)	49.17 (5.22)	1.67 (0.92)	1.44 (0.73)	/	/	Duloxetine + oral NSAIDs	NSAIDs	/	knee	VAS WOMAC
16	Li 2024^9^	Pain Physician	10.36076/ppj.2024.27.E45	China	NCT04117893	75 (47/28)	75 (39/36)	64 (6)	62.3 (7.2)	6.4 (2.8)	6.7 (3.6)	25.4 (3.4)	25.7 (4)	Duloxetine + HA + CS	HA + CS	/	Knee	BPI-S ratings BPI-I ratings 24-h average pain score 24-h worst pain score BPI average pain WOMAC PGI VAS HADS-D (Depression) HADS-A (Anxiety) AEs
17	Illeez 2022^38^	Rev Assoc Med Bras	10.1590/1806-9282.20211047	Turkey	/	30	30	/	/	/	/	/	/	Duloxetine	Pregabalin	/	knee	VAS WOMAC SF-36
18	Li 2024^25^	Contemporary Chinese Medicine	/	China	/	75 (33/42)	75 (30/45)	61.92 (10.83)	/	5.15 (1.78)	5.38 (1.84)	/	/	Duloxetine + NSAIDs	NSAIDs	/	knee	SF-36 VAS HAMA AEs
19	Wang 2018^28^	Chinese Journal of Pain Medicine	/	China	/	40 (11/29)	40 (12/28)	65.3 (4.6)	65.7 (5.1)	/	/	22.9 (2.2)	23.1 (1.9)	Duloxetine	Celecoxib	8 W	knee	WOMAC VAS AEs
20	Hu 2023^29^	Chinese Journal of Orthopaedics and Traumatology	/	China	/	60 (18/42)	58 (19/39)	65.42 (2.17)	63.32 (2.29)	8.17 (1.63)	8.20 (1.28)	/	/	Duloxetine	Meloxicam	6 M	knee	VAS

AEs, adverse events; AUSCAN, Australian/Canadian Hand Osteoarthritis Index; BMI, body-mass index; BPI-I, Brief Pain Inventory-Interference; BPI-S, Brief Pain Inventory-Severity; CGI-S, Clinical Global Impression-Severity; CS, corticosteroid; DOI, digital object identifier; EQ-5D-5L, EuroQol 5-Dimension 5-Level; HADS-A, Hospital Anxiety and Depression Scale-Anxiety; HADS-D, Hospital Anxiety and Depression Scale-Depression; HA, hyaluronic acid; HAMA, Hamilton Anxiety Rating Scale; HAMD, Hamilton Depression Rating Scale; NCT, ClinicalTrials.gov identifier; NRS, numerical rating scale; NSAIDs, non-steroidal anti-inflammatory drugs; OA, osteoarthritis; PGI-I, Patient Global Impression of Improvement; SAEs, serious adverse events; SD, standard deviation; SF-36, 36-Item Short Form Health Survey; VAS, visual analogue scale; WOMAC, Western Ontario and McMaster Universities Osteoarthritis Index.

Data are n (men/women) or mean (SD), unless otherwise stated. W, weeks; M, months; NR, not reported.

Risk-of-bias assessments varied across the included trials. Random sequence generation was assessed as having a low risk of bias in 14 studies, incomplete outcome data in 15, and selective reporting in 17. Blinding of outcome assessment was adequate in several studies, but some remained unclear (Fig. 1b and c).

For pain outcomes, the number of contributing RCTs and participants for each pooled analysis is reported in the GRADE evidence profile. Across included RCTs, duloxetine was associated with reduced pain intensity across multiple validated measures. Significant benefits were observed in WOMAC total scores (SMD −0.74, 95% CI −1.43 to −0.04; low-certainty evidence) and WOMAC pain scores (−0.36, −0.58 to −0.14; low-certainty evidence), several BPI-S domains including worst pain (−0.40,–0.45 to −0.35; moderate-certainty evidence), least pain (−0.30, −0.41 to −0.20; moderate-certainty evidence), and pain right now (−0.33, −0.42 to −0.24; moderate-certainty evidence), as well as in response for pain reduction of 30% or more (RR 1.35, 95% CI 1.27–1.42) and 50% or more (1.54, 1.34–1.77). Duloxetine also produced significant improvements in 24-h worst pain (SMD −0.34, 95% CI −0.60 to −0.08; moderate-certainty evidence), VAS (−0.66, −1.20 to −0.12; low-certainty evidence). For NRS pain, the pooled estimate was imprecise and did not show a statistically significant difference (SMD −1.75, 95% CI −6.98 to 3.47; low-certainty evidence; Fig. 2). For AUSCAN outcomes, no statistically significant differences were observed for pain (SMD 0.32, 95% CI −2.99 to 3.64), stiffness (−0.06, −2.98 to 2.87), or function (0.24, −5.00 to 5.48; Supplementary Figure S1a).

**Fig. 2:**
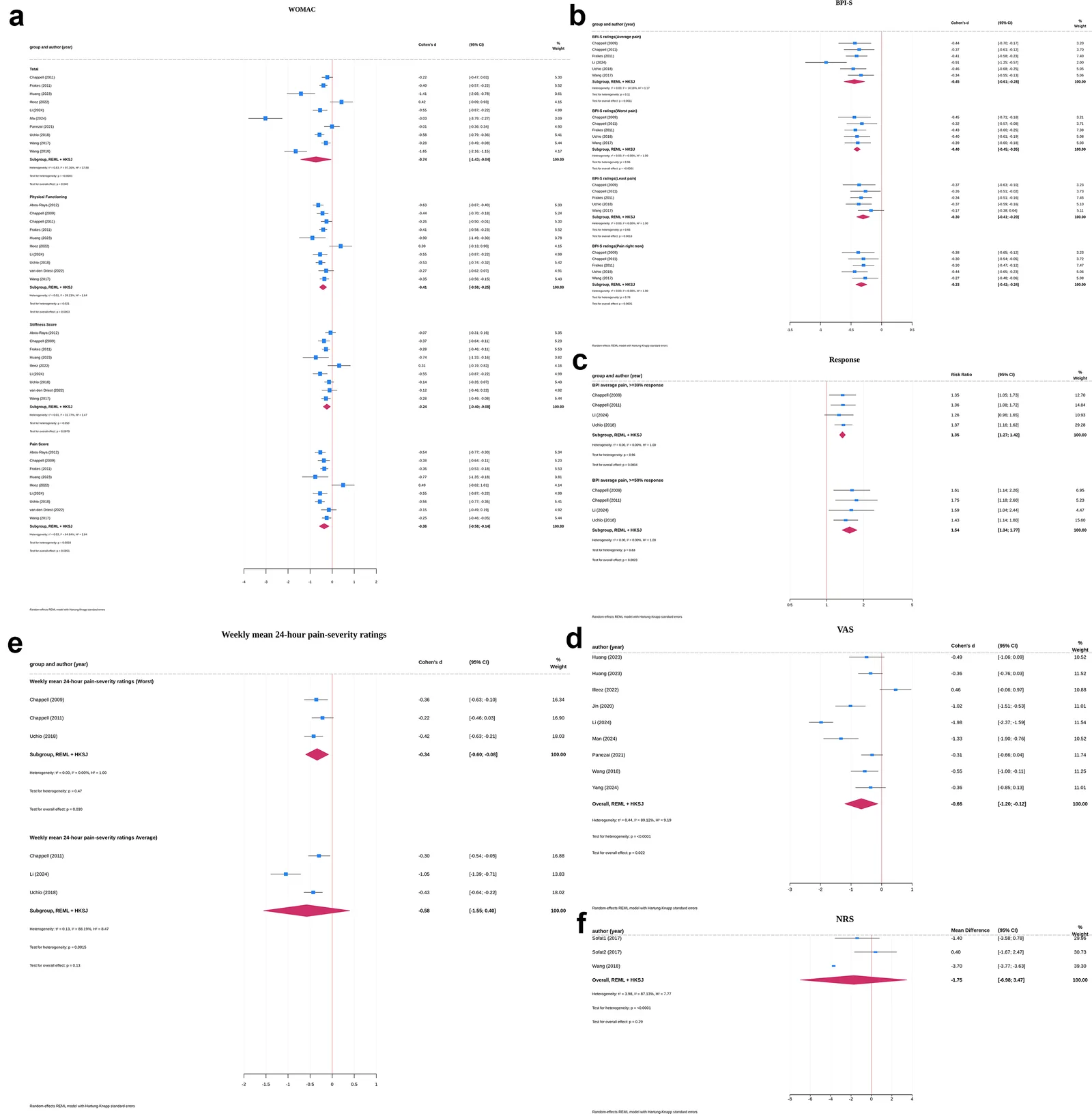
**Overall effects of duloxetine on pain-related outcomes in osteoarthritis.** Forest plots showing the pooled effects of duloxetine versus control interventions on (a) WOMAC total score, physical function, stiffness, and pain subscales; (b) BPI-Severity (BPI–S) domains, including average pain, worst pain, least pain, and pain right now; (c) responder outcomes defined as ≥30% and ≥50% reduction in BPI average pain; (d) weekly mean 24-h pain-severity ratings, including average and worst pain; (e) visual analogue scale (VAS) pain scores; and (f) numerical rating scale (NRS) pain scores. Negative standardised mean differences favour duloxetine for continuous pain outcomes. Risk ratios greater than 1 favour duloxetine for responder outcomes. Abbreviations: BPI-S, Brief Pain Inventory-Severity; NRS, numerical rating scale; VAS, visual analogue scale; WOMAC, Western Ontario and McMaster Universities Osteoarthritis Index.

For function-related outcomes, duloxetine was associated with significant improvements in physical function and stiffness. In the WOMAC subscales, physical function scores showed a moderate benefit (SMD −0.41, 95% CI −0.58 to −0.25), while stiffness scores improved modestly (−0.24, −0.40 to −0.08; Fig. 2a). Consistent improvements were also observed across multiple domains of the BPI-I (Fig. 3a), including general activity (SMD −0.36, 95% CI −0.48 to −0.23), normal work (−0.33, −0.49 to −0.17), mood (−0.29, −0.42 to −0.16), walking ability (−0.34, −0.48 to −0.20), sleep (−0.28, −0.45 to −0.10), and enjoyment of life (−0.29, −0.57 to −0.02). By contrast, the AUSCAN outcomes did not demonstrate significant benefits. Both stiffness (SMD −0.06, 95% CI −2.98 to 2.87) and function (0.24, −5.00 to 5.48) failed to show improvements compared with controls (Supplementary Figure S1a, Table 1).

**Fig. 3:**
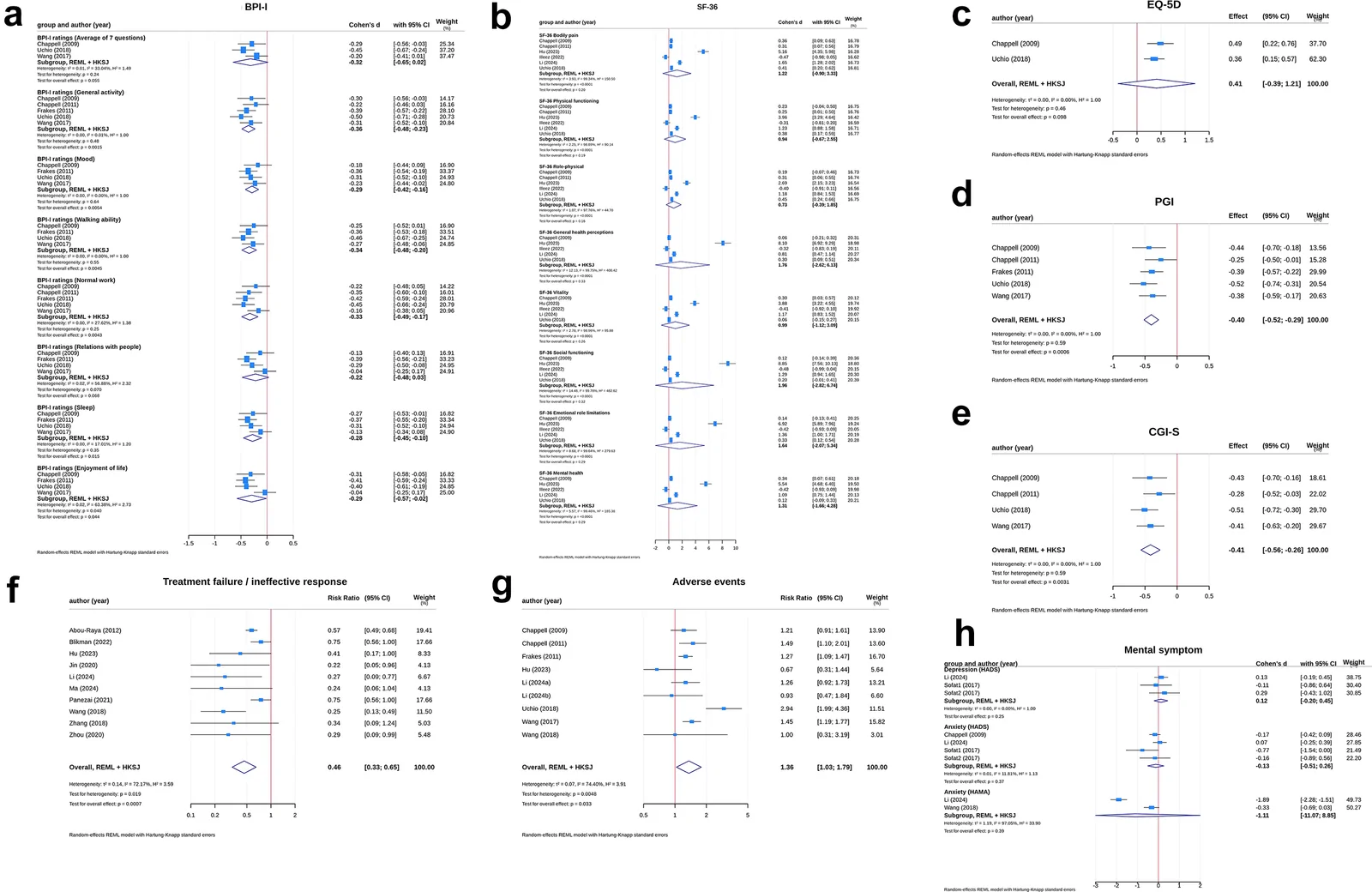
**Overall effects of duloxetine on functional interference, quality of life, global impression, treatment failure, mental health, and safety outcomes.** (a) Forest plots of BPI-Interference domains. (b) Forest plots of SF-36 quality-of-life domains. (c) Forest plot of EQ-5D. (d) Forest plot of PGI-I. (e) Forest plot of CGI-S. (f) Forest plot of treatment failure/ineffective response. (g) Forest plot of adverse events. (h) Forest plots of mental health outcomes, including depression and anxiety measures. Negative standardised mean differences favour duloxetine for symptom outcomes. For treatment failure/ineffective response, risk ratios less than 1 favour duloxetine. For adverse events, risk ratios greater than 1 indicate a higher risk in the duloxetine group. Abbreviations: BPI-I, Brief Pain Inventory-Interference; CGI-S, Clinical Global Impression-Severity; EQ-5D, EuroQol 5-Dimension; PGI-I, Patient Global Impression of Improvement; SF-36, 36-Item Short Form Health Survey.

For health-related quality-of-life, global impression, psychological, and safety outcomes, pooled SF-36 estimates were imprecise and should be interpreted cautiously. The SMDs were 1.22 (95% CI −0.90 to 3.33) for bodily pain, 0.94 (−0.67 to 2.55) for physical functioning, 0.73 (−0.39 to 1.85) for role limitations due to physical problems, 1.76 (−2.62 to 6.13) for general health perceptions, 0.99 (−1.12 to 3.09) for vitality, 1.96 (−2.82 to 6.74) for social functioning, 1.64 (−2.07 to 5.34) for role limitations due to emotional problems, and 1.31 (−1.66 to 4.28) for mental health (Fig. 3b; moderate-certainty evidence). The EQ-5D estimate was not statistically significant (SMD 0.41, 95% CI −0.39 to 1.21; moderate-certainty evidence). Global impression measures supported these findings, with significant improvements in the PGI-I overall (SMD −0.40, 95% CI −0.52 to −0.29; moderate-certainty evidence) and CGI-S (−0.41, −0.56 to −0.26; moderate-certainty evidence; Fig. 3c–e). Across the included trials, duloxetine was associated with a significantly lower risk of treatment failure compared with control interventions (RR 0.46, 95% CI = 0.33–0.65; low-certainty evidence; Fig. 3f). Because heterogeneity was extremely high across the SF-36 analyses, the pooled estimates have limited clinical interpretability and should be interpreted with considerable caution. None of the SF-36 domains showed a statistically significant pooled effect.

Psychological outcomes were analysed separately for depression and anxiety. There was no clear evidence of an effect on depression (SMD 0.12, 95% CI −0.20 to 0.45; moderate-certainty evidence). For anxiety, neither HADS-A (SMD −0.13, −0.51 to 0.26; moderate-certainty evidence) nor HAMA (−1.11, −11.07 to 8.85; low-certainty evidence) showed a clear treatment effect. Relatively few studies reported depression- or anxiety-specific symptom scales suitable for quantitative synthesis. Broader mood-related or mental-health-related domains, including SF-36 mental health and BPI-I mood, were analysed under their corresponding parent instruments rather than pooled with depression/anxiety-specific scales. Regarding safety, duloxetine was associated with a significantly higher risk of adverse events (RR 1.36, 95% CI 1.03–1.79; low-certainty evidence; Fig. 3g and h). However, this finding should be interpreted cautiously because the certainty of evidence was low and the estimate was imprecise.

Publication bias and the influence of individual studies were assessed for prespecified outcomes. Funnel plots of WOMAC, VAS, and adverse events are presented in Supplementary Figure S1b–d. Egger's test suggested potential funnel plot asymmetry for WOMAC (p = 0.014), whereas no significant asymmetry was detected for VAS (p = 0.84) or adverse events (p = 0.27). Therefore, although the findings for VAS and adverse events did not indicate significant publication bias, the WOMAC results should be interpreted with caution and were further assessed using sensitivity analyses.

Given the high heterogeneity observed in the initial analysis (WOMAC-total/Physical function/pain score), we conducted a sensitivity analysis to assess the impact of individual studies on the results. After excluding influential studies identified through leave-one-out sensitivity analyses,^32^, ^38^ heterogeneity was reduced for physical function and WOMAC pain (physical function: I^2^ from 39.13% to 5.71%; WOMAC pain: I^2^ from 64.84% to 27.03%), whereas substantial heterogeneity persisted for WOMAC total score after exclusion of Ma 2024 (I^2^ from 97.36% to 93.95%). The pooled estimates remained statistically significant after exclusion (WOMAC total SMD −0.50, −0.98 to −0.02; physical function SMD −0.44, −0.55 to −0.33; WOMAC pain SMD −0.41, −0.54 to −0.28). These findings should be interpreted cautiously, particularly for WOMAC total score, because substantial residual heterogeneity remained.

Exploratory meta-regression analyses were conducted to investigate whether maintenance dose, treatment duration, and study quality contributed to heterogeneity in WOMAC outcomes (Supplementary Table S2). No statistically significant associations were identified between treatment effect and maintenance dose, treatment duration, or study quality for WOMAC total, pain, physical function, or stiffness outcomes. However, the relatively small number of studies contributing to these analyses limited the statistical power to detect effect modification. The study-level covariates accounted for 0–13.9% of the between-study heterogeneity, and these estimates should therefore be interpreted cautiously.

Subgroup analyses stratified by treatment duration (<12 weeks versus ≥12 weeks) were performed for WOMAC outcomes (Supplementary Table S3). No statistically significant pooled effects were observed in the short-term subgroup. In the long-term subgroup, WOMAC pain, physical function, and stiffness showed statistically significant pooled estimates, whereas WOMAC total score did not. No significant differences were observed between duration subgroups for WOMAC total scores (p = 0.34), pain scores (p = 0.51), physical function (p = 0.46), or stiffness (p = 0.34). Because the small number of studies within each subgroup limited statistical power, these findings should be regarded as exploratory.

The certainty of evidence for outcomes was assessed using the GRADE framework (Supplementary Table S1). Evidence certainty ranged from low to high across outcomes. Low-certainty evidence was observed for WOMAC total scores, WOMAC pain scores, VAS, NRS, BPI-I average score, treatment failure/ineffective response, HAMA, and overall adverse events, primarily due to concerns regarding risk of bias, inconsistency, and imprecision. Moderate-certainty evidence supported WOMAC physical function and stiffness, BPI-S pain domains, weekly 24-h worst-pain ratings, most BPI-I domains, SF-36 domains, EQ-5D, PGI-I, CGI-I, and HADS depression and anxiety outcomes. High-certainty evidence was observed for several pain-related outcomes, including responder analyses (≥30% and ≥50% pain reduction). Overall, downgrading was mainly attributable to study limitations, between-study heterogeneity, and imprecision.

Comparator-stratified analyses were conducted separately for placebo-controlled, active-comparator, and add-on/background-therapy settings, which addressed distinct clinical questions.

In placebo-controlled studies, the number of contributing RCTs and participants for each pooled analysis is reported in the GRADE evidence profile. In the placebo-controlled subgroup, duloxetine consistently demonstrated significant benefits across core outcomes. WOMAC scores showed moderate improvements in the total score (SMD –0.38, 95% CI −0.62 to −0.14; I^2^ = 47.38%), physical functioning (−0.44,–0.57 to −0.30; I^2^ = 11.83%), stiffness (−0.23,–0.37 to −0.10; I^2^ = 0.01%), and pain subscales (−0.41,–0.57 to −0.25; I^2^ = 31.37%), as well as in response for pain reduction of 30% or more (RR 1.22, 95% CI 1.20–1.23) and 50% or more (1.36, 1.08–1.72; Fig. 4a).

**Fig. 4:**
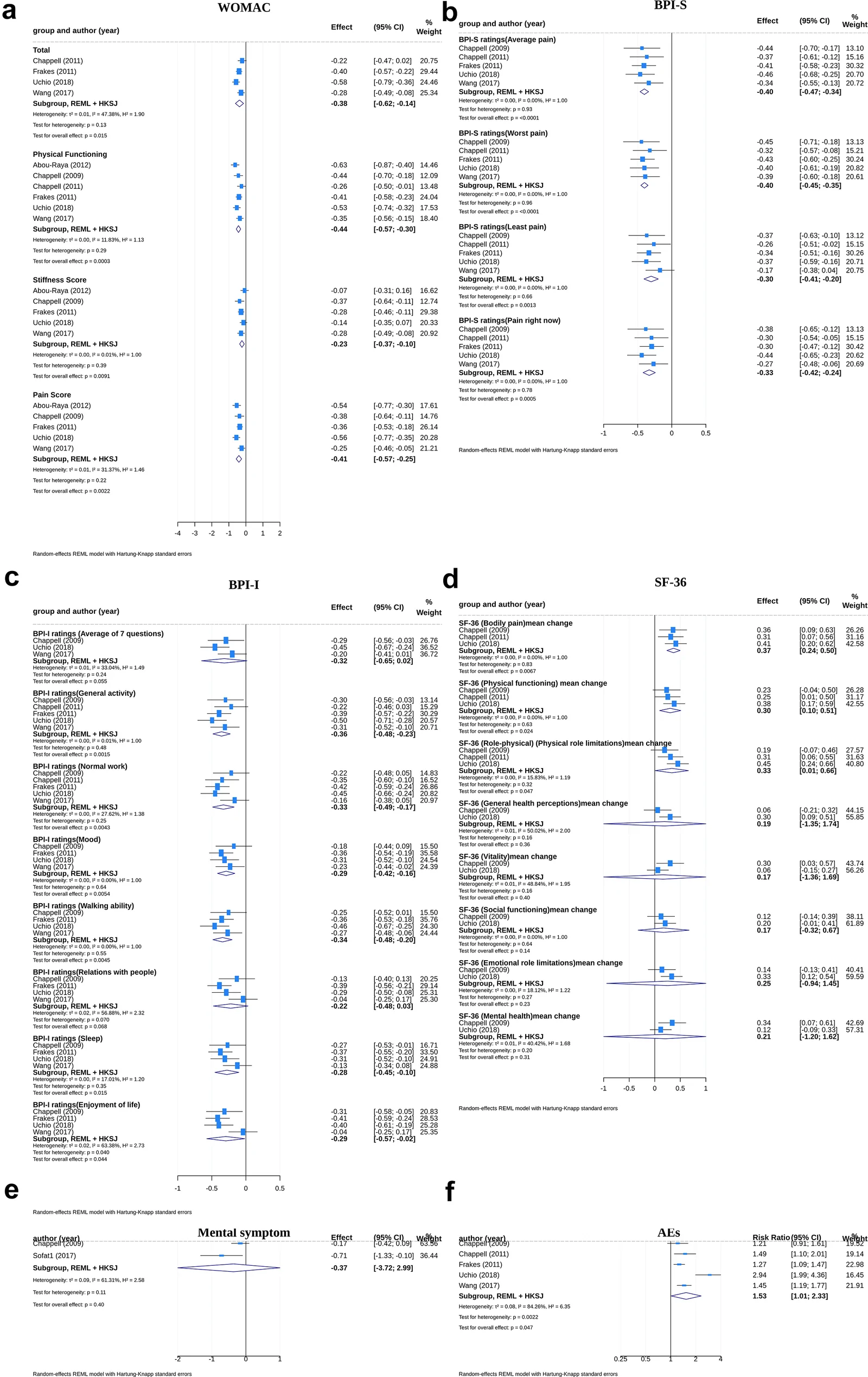
**Duloxetine versus placebo.** (a) Forest plots of WOMAC outcomes. (b) Forest plots of BPI-Severity domains. (c) Forest plots of BPI-Interference domains. (d) Forest plots of SF-36 quality-of-life outcomes. (e) Forest plot of anxiety outcomes. (f) Forest plot of adverse events. Abbreviations: BPI-I, Brief Pain Inventory-Interference; BPI-S, Brief Pain Inventory-Severity; SF-36, 36-Item Short Form Health Survey; WOMAC, Western Ontario and McMaster Universities Osteoarthritis Index.

Pain reduction was further supported by BPI-S outcomes. Duloxetine significantly improved average pain (SMD −0.40, 95% CI −0.47 to −0.34), worst pain (−0.40, −0.45 to −0.35), least pain (−0.30, −0.41 to −0.20), and pain right now (−0.33, −0.42 to −0.24), all with I^2^ of 0.0% (Fig. 4b). BPI-I domains also improved, including general activity (SMD –0.36, 95% CI −0.48 to −0.23), normal work (−0.33, −0.49 to −0.17), mood (−0.29, −0.42 to −0.16), walking ability (−0.34, −0.48 to −0.20), sleep (−0.28, −0.45 to −0.10), and enjoyment of life (−0.29, −0.57 to −0.02). The effects on relations with people (SMD −0.22, 95% CI −0.48 to 0.03) and the average BPI-I score (−0.32, −0.65 to 0.02) were not statistically significant (Fig. 4c). Health-related quality of life also improved, including SF-36 domains such as bodily pain (SMD 0.37, 95% CI 0.24–0.50), physical functioning (0.30, 0.10–0.51), and role limitations due to physical problems (0.33, 0.01–0.66). Other SF-36 domains showed no statistically significant differences, including general health perception, vitality, social functioning, role limitations due to emotional problems, and mental health (Fig. 4d).

In addition, no statistically significant difference in anxiety symptoms was observed (SMD −0.37, 95% CI −3.72 to 2.99; Fig. 4e). However, duloxetine was also associated with a significantly higher risk of adverse events compared with placebo (RR 1.53, 95% CI 1.01–2.33; Fig. 4f). Overall, placebo-controlled studies provided evidence of improvements in pain and function, with statistically significant differences in selected SF-36 domains.

In active-comparator studies, the active-comparator subgroup included pregabalin, celecoxib, meloxicam, NSAID-based treatment, and usual care as comparators. Where reported, comparator doses included pregabalin 300 mg/day and celecoxib 100–200 mg/day; doses were not consistently reported for all active comparators. Outcome availability differed across studies: WOMAC total score was reported in three studies, VAS in four, and SF-36 in two. In comparison with active pharmacological comparators, duloxetine did not show a statistically significant advantage for WOMAC total score (SMD −1.40, −5.71 to 2.90; I^2^ = 97.08%) or VAS pain score (−0.44, −1.60 to 0.72; I^2^ = 87.32%; Fig. 5a and b). Individual study results varied, and the pooled estimates were imprecise and showed substantial between-study heterogeneity.

**Fig. 5:**
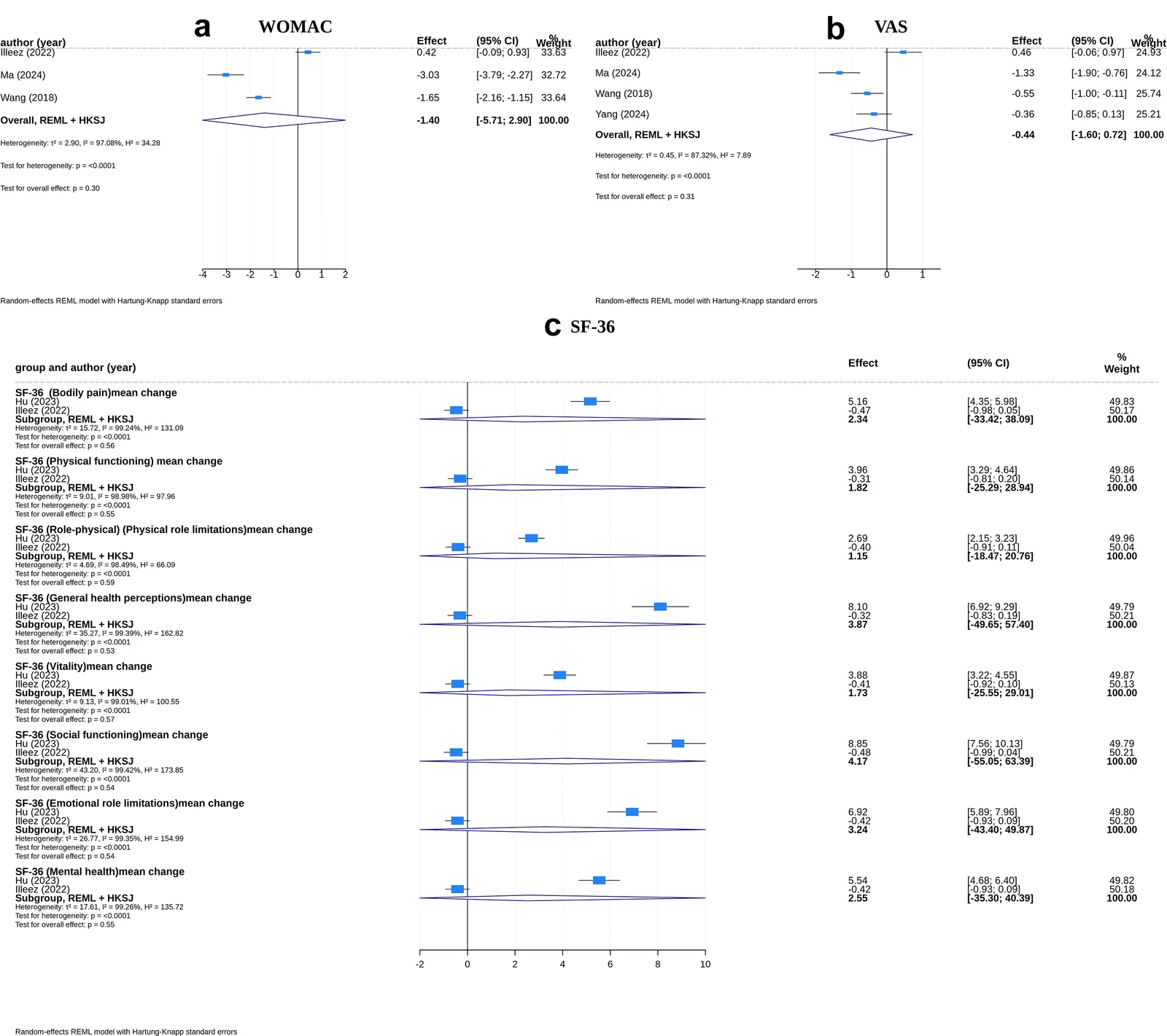
**Duloxetine versus active pharmacological comparators.** (a) Forest plots of WOMAC outcomes. (b) Forest plot of VAS pain outcomes. (c) Forest plots of SF-36 quality-of-life domains. Estimates should be interpreted with caution because of substantial between-study heterogeneity. Abbreviations: SF-36, 36-Item Short Form Health Survey; VAS, visual analogue scale; WOMAC, Western Ontario and McMaster Universities Osteoarthritis Index.

Quality-of-life outcomes measured by the SF-36 showed no statistically significant pooled effects across all assessed domains, including bodily pain (SMD 2.34, 95% CI −33.42 to 38.09), physical functioning (1.82, −25.29 to 28.94), role limitations due to physical problems (1.15, −18.47 to 20.76), general health perceptions (3.87, −49.65 to 57.40), vitality (1.73, −25.55 to 29.01), social functioning (4.17, −55.05 to 63.39), role limitations due to emotional problems (3.24, −43.40 to 49.87), and mental health (2.55, −35.30 to 40.39; Fig. 5c). Heterogeneity was extremely high across SF-36 domains, and the confidence intervals were very wide, indicating substantial uncertainty. Overall, duloxetine did not demonstrate clear superiority over active pharmacological comparators for pain, function, or quality-of-life outcomes. Given the small number of studies, substantial between-study heterogeneity, and differences in active pharmacological comparators, these pooled estimates should be regarded as descriptive summaries of the available evidence rather than as estimates of a single common treatment effect.

In add-on/background-therapy studies, background or concomitant treatments included radiofrequency therapy, surgery, usual care, NSAIDs, loxoprofen plus glucosamine, and intra-articular hyaluronic acid plus corticosteroid injection. These studies addressed the broader clinical question of whether duloxetine provides additional benefit when added to background treatment, but the background interventions differed substantially; therefore, this subgroup was considered exploratory. For WOMAC outcomes, the add-on/background therapy analyses were based on three studies with 326–330 participants. The pooled SMDs were −0.62 (95% CI −2.34 to 1.10; I^2^ = 90.53%) for total score, −0.51 (−1.19 to 0.18; I^2^ = 38.55%) for physical functioning, −0.43 (−1.20 to 0.35; I^2^ = 57.73%) for stiffness, and −0.44 (−1.19 to 0.30; I^2^ = 53.70%) for pain score (Fig. 6a). Four studies with 398 participants informed VAS pain scores; the pooled estimate was imprecise, with a confidence interval crossing the null (SMD −0.95, 95% CI −2.16 to 0.25; I^2^ = 91.44%; Fig. 6b). However, there was no evidence of improvement in anxiety symptoms as measured by HAMA (SMD –1.11, 95% CI –11.07 to 8.85; I^2^ = 97.05%; Fig. 6c). Safety analyses showed no statistically significant difference in adverse events between the combination therapy and control groups (RR 1.20, 95% CI 0.27–5.34; I^2^ = 0.0%; Fig. 6d). However, the CI remained compatible with a clinically relevant increase in adverse-event risk, and this finding should not be interpreted as evidence of comparable safety. Treatment discontinuation due to adverse events was assessed as an additional tolerability outcome. Seven studies provided estimable data, and duloxetine was associated with a higher risk of treatment discontinuation due to adverse events than control treatment (RR 2.28, 95% CI 1.59–3.27; I^2^ = 0.0%). Across the add-on/background-therapy analyses, pooled estimates for WOMAC, VAS, HAMA, and adverse events were imprecise and crossed the null. Given the small number of studies, heterogeneous background treatments, substantial heterogeneity for several outcomes, and very-low-certainty evidence, these findings were inconclusive and do not establish a consistent additive treatment effect (Supplementary Table S5).

**Fig. 6:**
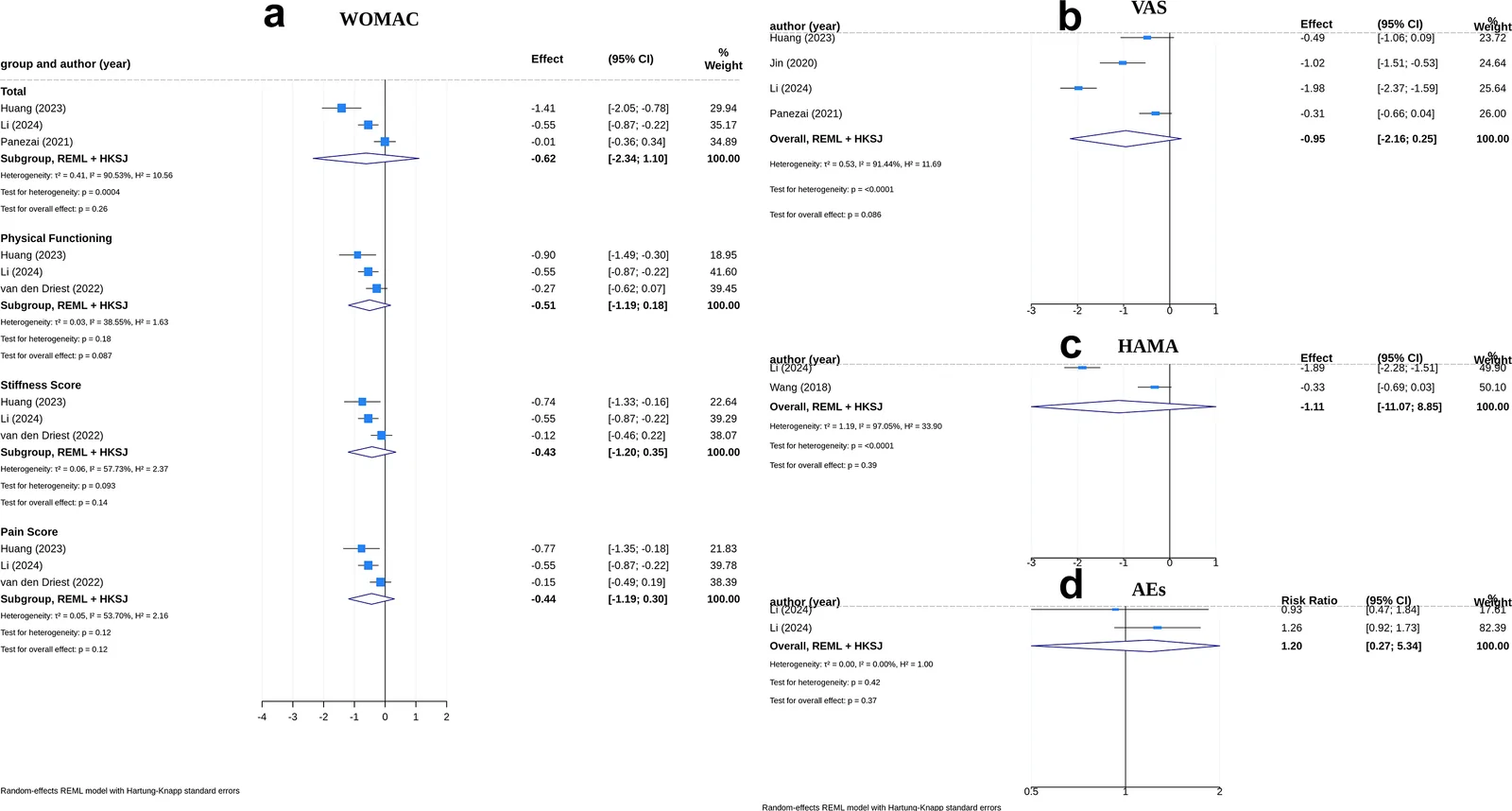
**Duloxetine add-on or combination therapy versus background therapy/control.** (a) Forest plots of WOMAC outcomes. (b) Forest plot of VAS pain outcomes. (c) Forest plot of anxiety outcomes. (d) Forest plot of adverse events. Abbreviations: HAMA, Hamilton Anxiety Rating Scale; VAS, visual analogue scale; WOMAC, Western Ontario and McMaster Universities Osteoarthritis Index.

## Discussion

The principal finding of this review is that the available evidence can be described separately according to comparator category. Placebo-controlled trials provided the clearest evidence of benefit, whereas active-comparator and add-on/background-therapy estimates were less precise and should be interpreted separately. These analyses do not provide a direct test of whether duloxetine effects differ between comparator categories. The clearest evidence was observed in placebo-controlled comparisons, whereas evidence of superiority over active pharmacological comparators was limited, and add-on or combination therapy evidence was inconclusive. In this study, pain relief and function were used as the primary outcome measures.^14^, ^39^ Duloxetine was associated with reductions in pain severity across several measures, including BPI-S, VAS and WOMAC pain, while BPI-I indicated improvements in pain-related interference. However, the magnitude and certainty of these effects varied across outcomes and treatment settings, and the findings should therefore be interpreted cautiously. Improving physical function is a core objective in the treatment of patients with osteoarthritis, as joint function is crucial for maintaining daily activities and quality of life. Our findings suggest that duloxetine was associated with improvements in physical function, particularly as assessed using the WOMAC physical function subscale. Improvements were also observed in several related outcomes, including stiffness, health-related quality of life, and global assessments, although these represent distinct outcome domains and the magnitude and precision of the estimates varied. Previous studies have similarly shown that improving physical function is key to managing osteoarthritis and that therapies targeting both pain and function are essential for improving patients’ overall well-being.^40^

Moreover, patients receiving duloxetine were significantly more likely to achieve the established responder thresholds of ≥30% and ≥50% pain relief.^41^ These responder outcomes provide a clinically interpretable measure of treatment benefit and complement the SMD-based analyses of continuous pain outcomes. However, they do not identify specific patient subgroups that are more likely to respond to duloxetine.

Although duloxetine has established analgesic and antidepressant effects in other clinical contexts,^1^, ^7^, ^8^, ^42^ we did not observe a significant improvement in depression outcomes among osteoarthritis patients treated with duloxetine. This finding should be interpreted cautiously because most included trials were not designed or powered to evaluate antidepressant effects, and participants were generally not selected based on clinically diagnosed depression. Therefore, the absence of a significant effect on depression in these osteoarthritis trials should not be interpreted as evidence that duloxetine lacks antidepressant benefit in osteoarthritis patients with comorbid depression. No statistically significant difference in anxiety symptoms was observed in the placebo-controlled subgroup; the estimate was imprecise and based on limited low-certainty evidence. Further studies specifically enrolling osteoarthritis patients with depression or anxiety are needed to clarify the psychological benefits of duloxetine and its potential role in addressing the psychological dimensions of chronic osteoarthritis pain.

This review synthesised placebo-controlled, active-comparator, and add-on/background-therapy evidence separately according to comparator type. Duloxetine was evaluated in placebo-controlled, active-comparator, and add-on or combination-therapy comparisons, allowing the available evidence to be considered separately according to the type of comparator. Increasing evidence suggests that osteoarthritis pain is multifactorial and involves not only peripheral nociceptive input from joint degeneration but also central sensitisation mechanisms that contribute to chronic pain persistence and symptom amplification.^43^, ^44^ As a serotonin–norepinephrine reuptake inhibitor, duloxetine modulates descending inhibitory pain pathways, this mechanism provides a biological rationale for investigating whether pain phenotype influences treatment response.^39^ However, the present analyses cannot determine whether differences between placebo-controlled and active-comparator findings are attributable to differences in underlying pain mechanisms or comparator effects. Whether patients with centrally mediated pain features, widespread pain, or other specific clinical phenotypes derive greater benefit from duloxetine remains uncertain and requires direct evaluation in appropriately designed studies. The available active-comparator evidence did not provide clear evidence that duloxetine was superior to the pharmacological comparators studied for pain or physical function. However, these analyses included relatively few studies and showed substantial heterogeneity for several outcomes. The available active-comparator evidence did not show a significant advantage of duloxetine for WOMAC total score or VAS pain score, with substantial heterogeneity across studies. For SF-36 quality-of-life domains, confidence intervals were wide and crossed the null, and the high heterogeneity suggests these findings should be interpreted with caution. Evidence for duloxetine as add-on or combination therapy was based on only three studies involving 330 participants, with differences in background treatments and comparator interventions. The pooled estimates for pain, physical functioning, and stiffness were imprecise and findings were not consistent across individual comparisons including non-significant findings in comparisons with usual care. Accordingly, these pooled estimates should be considered exploratory rather than evidence of a consistent additive treatment effect. Although no statistically significant difference in adverse events was detected, the confidence interval was compatible with a clinically relevant increase in risk. The available evidence therefore does not establish comparable safety between treatment groups.^45^, ^46^, ^47^ The supplementary analysis of treatment discontinuation due to adverse events further suggested that duloxetine may be less well tolerated than control treatment in some settings. However, because the number of contributing studies was limited and comparator settings varied, this finding should be interpreted cautiously and should not be considered definitive evidence of comparative safety. In addition, no clear improvement in anxiety symptoms was observed in the combination group; however, the limited number of contributing studies and imprecision of the estimates preclude firm conclusions regarding psychological outcomes. Further research is needed to elucidate the mechanisms and clinical implications of such combinations, including potential synergistic effects or altered side–effect profiles.

The present findings may also have implications for the interpretation of current clinical practice guidelines. Current guidelines generally recommend duloxetine as a conditional pharmacological option for patients with persistent pain, particularly when first-line therapies such as NSAIDs are insufficient, contraindicated, or poorly tolerated.^4^, ^48^ Our findings are broadly consistent with evidence of symptomatic benefit compared with placebo, whereas comparative effectiveness against active pharmacological treatments remains uncertain. The inconclusive add-on or combination-therapy estimates do not establish additional symptomatic benefit when duloxetine is used alongside conventional analgesics; however, the present meta-analysis cannot determine which patient phenotypes are most likely to benefit. Evidence for add-on or combination therapy was limited and heterogeneous, and the small number of studies and participants precludes firm conclusions regarding either an additive treatment effect or the optimal clinical use of duloxetine in combination therapy. Future trials should directly compare duloxetine with established active treatments and evaluate whether patient characteristics or pain phenotypes modify treatment response.

The GRADE assessment provides additional context for interpreting the present findings. The certainty of evidence varied across outcomes. Evidence supporting improvements in WOMAC pain, total scores, and overall adverse events was rated as low certainty, whereas evidence for WOMAC physical function and stiffness was of moderate certainty. Several secondary outcomes were supported by moderate-certainty evidence, including BPI-S measures, most BPI-I domains, SF-36 domains, EQ-5D, PGI-I, CGI-I, and HADS depression and anxiety outcomes. High-certainty evidence was observed only for responder outcomes, including ≥30% and ≥50% pain reduction. The primary reasons for downgrading the certainty of evidence were methodological limitations and between-study heterogeneity. Therefore, the magnitude of benefit for several outcomes remains uncertain and should be interpreted in accordance with the corresponding certainty of evidence.

Meta-regression did not identify statistically significant associations between maintenance dose, treatment duration, or study quality and WOMAC treatment effects. However, the relatively small number of studies limited the power to detect effect modification, and these findings should not be interpreted as evidence that these factors do not influence treatment response. Other factors, such as patient characteristics, osteoarthritis severity, pain phenotype, concomitant therapies, or differences in study populations, may have contributed to the residual heterogeneity observed in several analyses. Subgroup analyses based on treatment duration did not identify significant differences between short-term (<12 weeks) and long-term (≥12 weeks) studies. The limited number of studies reduces the power to detect subgroup differences. These findings should therefore not be interpreted as evidence that treatment duration does not modify the effect of duloxetine.

This review has several limitations. Although sensitivity analyses, meta-regression analyses, and duration-based subgroup analyses were used to explore the consistency and heterogeneity of the findings, substantial heterogeneity remained for several WOMAC and comparator-stratified outcomes. Meta-regression did not identify statistically significant associations between maintenance dose, treatment duration, or study quality and WOMAC treatment effects. However, the relatively small number of studies limited the power to detect effect modification, and these findings should not be interpreted as evidence that these factors do not influence treatment response. Although leave-one-out analyses showed that the direction of the pooled estimates was not materially altered by exclusion of influential studies, substantial residual heterogeneity remained for some outcomes. Therefore, these sensitivity analyses indicate directional consistency for some WOMAC outcomes, but they do not establish clinical homogeneity or certainty in the magnitude of the pooled effects, particularly for WOMAC total. This heterogeneity may partly reflect differences in study populations, osteoarthritis severity, affected joints, treatment regimens, and comparator interventions, which ranged from placebo to active pharmacological treatments and combination therapies. Comparator stratification reduced, but did not eliminate, clinical heterogeneity. Active-comparator studies involved different pharmacological agents, while add-on or combination-therapy studies differed in background and concomitant treatments. For analyses with very high statistical heterogeneity, particularly the active-comparator SF-36 outcomes, quantitative pooling provides only a descriptive summary of the available evidence and should not be interpreted as evidence of a common treatment effect across studies. Differences in osteoarthritis site and severity, baseline pain intensity, symptom duration, pain phenotype, duloxetine dosing and titration, and concomitant or background therapy may have contributed to the observed clinical heterogeneity. These characteristics were incompletely or inconsistently reported across studies, and the small number of studies within individual categories limited our ability to examine these potential effect modifiers reliably in additional subgroup analyses. Pain phenotype was not evaluated as an effect modifier; therefore, this review cannot determine whether centrally mediated pain or other specific pain phenotypes identify patients who are more likely to benefit from duloxetine. Accordingly, the pooled effect estimates should be interpreted with appropriate caution. Several comparator-stratified analyses included only a small number of studies. In particular, the add-on or combination-therapy evidence comprised only three studies involving 330 participants, with differences in background treatments and comparator interventions and inconsistency across individual comparisons, including non-significant findings versus usual care. Consequently, these pooled estimates are imprecise and should be regarded as exploratory rather than as evidence of a consistent additive treatment effect. Although we added a supplementary analysis of treatment discontinuation due to adverse events, reporting of this outcome was inconsistent across trials, and subgroup analyses by comparator type were not reliable because of the small number of studies. Additional adequately powered head-to-head and add-on trials are needed to strengthen the comparative evidence. In addition, most included studies focused on knee osteoarthritis, whereas evidence for hand and hip osteoarthritis was limited, which may restrict the generalisability of the findings across all osteoarthritis phenotypes. Relatively few studies reported depression and anxiety outcomes, and inconsistencies in assessment tools, adverse-event reporting, and outcome definitions limited the strength of these analyses. Only one study examined duloxetine treatment in osteoarthritis patients with diagnosed depression, but it was excluded because only one participant completed follow-up.^46^ Finally, one multi-arm study contributed more than one comparison because they contained multiple control groups, and several studies had unclear or high risk of bias in domains such as allocation concealment and blinding, which may have influenced the precision and certainty of the pooled estimates.

In conclusion, duloxetine was associated with modest improvements in pain and physical function in patients with osteoarthritis, with the clearest evidence observed in placebo-controlled comparisons. Evidence from active-comparator and add-on/background-therapy studies was limited, heterogeneous, and inconclusive; it does not establish superiority over active treatment or a consistent additive treatment effect. These findings support duloxetine as a conditional pharmacological option within multimodal osteoarthritis pain management, particularly when NSAIDs are unsuitable or poorly tolerated. Future trials should evaluate longer-term effectiveness, treatment discontinuation, safety, and potential effect modifiers using adequately powered comparative designs.

## Contributors

DZ and DJH conceived and designed the study. DZ, AS, BX, and GGZ conducted the literature search, screened the studies, and extracted the data. DZ, with input from AS and VV, analysed and interpreted the data. DZ draughted the manuscript, with critical input from LAD and DJH. DZ and DJH accessed and verified the extracted data and analytic dataset. All authors contributed to data interpretation, critically revised the manuscript for important intellectual content, read and approved the final version of the manuscript. DZ and DJH were responsible for the decision to submit the manuscript for publication.

## Data sharing statement

The extracted data and analytic code are available from the corresponding author on reasonable request.

## Declaration of interests

DJH is the editor of the osteoarthritis section for UpToDate and co-Editor in Chief of Osteoarthritis and Cartilage. DJH provides consulting advice on scientific advisory boards for Lilly, Sanofi, Novartis, Haleon and Tissuegene. All other authors declare no competing interests.
