# Supplementary material for: Efficacy and safety of duloxetine for osteoarthritis: a comparator-stratified systematic review and meta-analysis
Source: eClinicalMedicine. 2026 Oct 29;101:104296. doi: 10.1016/j.eclinm.2026.104296 (PMC13742335; doi:10.1016/j.eclinm.2026.104296)
Supplement: Supplementary file 1 [file mmc1.docx]

| **Supplementary Table S1. GRADE assessment of the certainty of evidence for efficacy and safety outcomes of duloxetine in OA** | | | | | | | | | | | | | | | | | | | | |
| --- | --- | --- | --- | --- | --- | --- | --- | --- | --- | --- | --- | --- | --- | --- | --- | --- | --- | --- | --- | --- |
| **Outcome** | **No of participants (studies)** | **Certainty Assessment** | | | | | | | | | | | | | **Effect estimate**  **(95% CI) †** | | | **GRADE*** | | |
|  |  | **Risk of Bias** | | **Inconsistency** | **Indirectness** | | | **Imprecision** | | **Other** | | | | |  |  |  |  |  |  |
| **WOMAC** | | | | | | | | | | | | | | | | | | | | |
| **Total** | **1863**  **(10 RCTs)** | **Serious** | | **Serious** | **Not serious** | | | **Not serious** | | **None** | | | | | **-0.74 (-1.43 to -0.04)** | | | ⨁⨁◯◯ **LOW** | | |
| **Physical functioning** | **1863**  **(10 RCTs)** | **Not serious** | | **Serious** | **Not serious** | | | **Not serious** | | **None** | | | | | **-0.41 (-0.58 to -0.25)** | | | ⨁⨁⨁◯  **Moderate** | | |
| **stiffness score** | **2222**  **(9 RCTs)** | **Serious** | | **Not Serious** | **Not serious** | | | **Not serious** | | **None** | | | | | **-0.24 (-0.40 to -0.08)** | | | ⨁⨁⨁◯ **Moderate** | | |
| **pain score** | **2222**  **(9 RCTs)** | **Serious** | | **Serious** | **Not serious** | | | **Not serious** | | **None** | | | | | **-0.36 (-0.58 to -0.14)** | | | ⨁⨁◯◯  **LOW** | | |
| **BPI-S** | | | | | | | | | | | | | | | | | | | | |
| **BPI-S ratings**  **(Average pain)** | **1741**  **(6 RCTs)** | **Not Serious** | | **Not serious** | **Not serious** | | | **serious** | | | **None** | | | | **-0.45 (-0.61 to -0.28)** | | | | ⨁⨁⨁◯ **Moderate** | |
| **BPI-S ratings**  **(Worst pain)** | **1591**  **(5 RCTs)** | **Not Serious** | | **Not serious** | **Not serious** | | | **serious** | | | **None** | | | | **-0.40 (-0.45 to -0.35)** | | | | ⨁⨁⨁◯ **Moderate** | |
| **BPI-S ratings**  **(Least pain)** | **1591**  **(5 RCTs)** | **Not Serious** | | **Not serious** | **Not serious** | | | **serious** | | | **None** | | | | **-0.30 (-0.41 to -0.20)** | | | | ⨁⨁⨁◯ **Moderate** | |
| **BPI-S ratings**  **(Pain right now)** | **1591**  **(5 RCTs)** | **Not Serious** | | **Not serious** | **Not serious** | | | **serious** | | | **None** | | | | **-0.33 (-0.42 to -0.24)** | | | | ⨁⨁⨁◯ **Moderate** | |
| **Response** | | | | | | | | | | | | | | | | | | | | |
| **BPI average pain(Weekly mean 24-hour average pain, % response≥30%)** | **1010**  **(4 RCTs)** | **Not Serious** | | **Not serious** | | **Not serious** | | | **Not serious** | | **None** | | | | | | **1.35 (1.27 to 1.42)** | | | ⨁⨁⨁⨁ **HIGH** |
| **BPI average pain(Weekly mean 24-hour average pain, % response≥50%)** | **1010**  **(4 RCTs)** | **Not Serious** | | **Not serious** | | **Not serious** | | | **Not serious** | | **None** | | | | | | **1.54 (1.34 to 1.77)** | | | ⨁⨁⨁⨁ **HIGH** |
| **24h Pain** | | | | | | | | | | | | | | | | | | | | |
| **Weekly mean 24-hour pain-severity ratings Average)** | **759**  **(3 RCTs)** | **Not Serious** | | **Serious** | **Not serious** | | **Serious** | | | | **None** | | | | | **-0.58 (-1.55 to 0.40)** | | | | ⨁⨁◯◯ **LOW** |
| **Weekly mean 24-hour pain-severity ratings (Worst)** | **759**  **(3 RCTs)** | **Not Serious** | | **Not serious** | **Not serious** | | **serious** | | | | **None** | | | | | **-0.34 (-0.60 to -0.08)** | | | | ⨁⨁⨁◯ **Moderate** |
| **VAS** | | | | | | | | | | | | | | | | | | | | |
| **VAS** | **796**  **(9 RCTs)** | **Serious** | | **Serious** | **Not serious** | | **Not serious** | | | | **None** | | | | | **-0.66 (-1.20 to -0.12)** | | | | ⨁⨁◯◯ **LOW** |
| **NRS** | | | | | | | | | | | | | | | | | | | | |
| **NRS** | **185**  **(2 RCTs)** | **Not Serious** | | **Serious** | **Not serious** | | **Serious** | | | | **None** | | | | | **-1.75 (-6.98 to 3.47)** | | | | ⨁⨁◯◯ **LOW** |
| **BPI-I ratings** | | | | | | | | | | | | | | | | | | | | |
| **BPI-I ratings**  **(General activity)** | **1771**  **(5 RCTs)** | **Not Serious** | | **Serious** | **Not serious** | | **Not serious** | | | | **None** | | | | | **-0.36 (-0.48 to -0.23)** | | | | ⨁⨁⨁◯ **Moderate** |
| **BPI-I ratings (Normal work)** | **1771**  **(5 RCTs)** | **Not Serious** | | **Serious** | **Not serious** | | **Not serious** | | | | **None** | | | | | **-0.33 (-0.49 to -0.17)** | | | | ⨁⨁⨁◯ **Moderate** |
| **BPI-I ratings(Mood)** | **1515**  **(4 RCTs)** | **Not Serious** | | **Serious** | **Not serious** | | **Not serious** | | | | **None** | | | | | **-0.29 (-0.42 to -0.16)** | | | | ⨁⨁⨁◯ **Moderate** |
| **BPI-I ratings (Walking ability)** | **1515**  **(4 RCTs)** | **Not Serious** | | **Serious** | **Not serious** | | **Not serious** | | | | **None** | | | | | **-0.34 (-0.48 to -0.20)** | | | | ⨁⨁⨁◯ **Moderate** |
| **BPI-I ratings(Relations with people)** | **1515**  **(4 RCTs)** | **Not Serious** | | **Not serious** | **Not serious** | | **serious** | | | | **None** | | | | | **-0.22 (-0.48 to 0.03)** | | | | ⨁⨁⨁◯ **Moderate** |
| **BPI-I ratings (Sleep)** | **1515**  **(4 RCTs)** | **Not Serious** | | **Serious** | **Not serious** | | **Not serious** | | | | **None** | | | | | **-0.28 (-0.45 to -0.10)** | | | | ⨁⨁⨁◯ **Moderate** |
| **BPI-I ratings(Enjoyment of life)** | **1515**  **(4 RCTs)** | **Not Serious** | | **Not serious** | **Not serious** | | **serious** | | | | **None** | | | | | **-0.29 (-0.57 to -0.02)** | | | | ⨁⨁⨁◯ **Moderate** |
| **BPI-I ratings (Average of 7 questions)** | **990**  **(3 RCTs)** | **Not Serious** | | **Serious** | **Not serious** | | **Serious** | | | | **None** | | | | **-0.32 (-0.65 to 0.02)** | | | | | ⨁⨁◯◯  **LOW** |
| **SF-36** | | | | | | | | | | | | | | | | | | | | |
| **SF-36 (Mental health)** | **1168**  **(6 RCTs)** | | **Not serious** | **Serious** | **Not serious** | | | **Not serious** | | | | **None** | | **1.31 (-1.66 to 4.28)** | | | | | | ⨁⨁⨁◯  **Moderate** |
| **SF-36 (Bodily pain)** | **1018**  **(6 RCTs)** | | **Not serious** | **Serious** | **Not serious** | | | **Not serious** | | | | **None** | | **1.22 (-0.9 to 3.33)** | | | | | | ⨁⨁⨁◯  **Moderate** |
| **SF-36 (Physical functioning)** | **1018**  **(6 RCTs)** | | **Not serious** | **Serious** | **Not serious** | | | **Not serious** | | | | **None** | | **0.94 (-0.67 to 2.55)** | | | | | | ⨁⨁⨁◯ **Moderate** |
| **SF-36 (Role-physical) (Physical role limitations)** | **1018**  **(6 RCTs)** | | **Not serious** | **Serious** | **Not serious** | | | **Not serious** | | | | **None** | | **0.73 (-0.39 to 1.85)** | | | | | | ⨁⨁⨁◯  **Moderate** |
| **SF-36 (General health perceptions)** | **912**  **(5 RCTs)** | | **Not serious** | **Serious** | **Not serious** | | | **Not serious** | | | | **None** | | **1.76 (-2.62 to 6.13)** | | | | | | ⨁⨁⨁◯  **Moderate** |
| **SF-36 (Vitality)** | **912**  **(5 RCTs)** | | **Not serious** | **Serious** | **Not serious** | | | **Not serious** | | | | **None** | | **0.99 (-1.12 to 3.09)** | | | | | | ⨁⨁⨁◯  **Moderate** |
| **SF-36 (Social functioning)** | **912**  **(5 RCTs)** | | **Not serious** | **Serious** | **Not serious** | | | **Not serious** | | | | **None** | | **1.96 (-2.82 to 6.74)** | | | | | | ⨁⨁⨁◯  **Moderate** |
| **SF-36 (Emotional role limitations)** | **912**  **(5 RCTs)** | | **Not serious** | **Serious** | **Not serious** | | | **Not serious** | | | | **None** | | **1.64 (-2.07 to 5.34)** | | | | | | ⨁⨁⨁◯  **Moderate** |
| **EQ-5D** | | | | | | | | | | | | | | | | | | | | |
| **EQ-5D** | **584**  **(2 RCTs)** | | **Not Serious** | **Not serious** | **Not serious** | | | **serious** | | | **None** | | **0.41 (-0.39 to 1.21)** | | | | | | | ⨁⨁⨁◯  **Moderate** |
| **PGI-I** | | | | | | | | | | | | | | | | | | | | |
| **PGI-I** | **1771**  **(5 RCTs)** | | **Not Serious** | **Not serious** | **Not serious** | | | **serious** | | | **None** | | **-0.40 (-0.52 to -0.29)** | | | | | | | ⨁⨁⨁◯  **Moderate** |
| **CGI-S** | | | | | | | | | | | | | | | | | | | | |
| **CGI-S** | **894**  **(4 RCTs)** | | **Not Serious** | **Not serious** | **Not serious** | | | **serious** | | | **None** | | **-0.41 (-0.56 to -0.26)** | | | | | | | ⨁⨁⨁◯  **Moderate** |
| **Mental symptom** | | | | | | | | | | | | | | | | | | | | |
| **Depression(Mean difference)-HADS** | **236**  **(3 RCTs)** | | **Not Serious** | **Not serious** | **Not serious** | | | **Serious** | | | **None** | | **0.12 (-0.20 to 0.45)** | | | | | | | ⨁⨁⨁◯ **Moderate** |
| **Anxiety(Mean difference)-HADS** | **467**  **(4 RCTs)** | | **Not Serious** | **Not serious** | **Not serious** | | | **serious** | | | **None** | | **-0.13 (-0.51 to 0.26)** | | | | | | | ⨁⨁⨁◯  **Moderate** |
| **HAMA** | **270**  **(2 RCTs)** | | **Not Serious** | **Serious** | **Not serious** | | | **serious** | | | **None** | | **-1.11 (-11.07 to 8.85)** | | | | | | | ⨁⨁◯◯  **LOW** |
| **Treatment failure/ineffective response** | | | | | | | | | | | | | | | | | | | | |
| **Treatment failure/ineffective response** | **1175**  **(10 RCTs)** | | **Serious** | **Serious** | **Not serious** | | | **Not serious** | | | | **None** | | **0.46 (0.33 to 0.65)** | | | | | | ⨁⨁◯◯  **LOW** |
| **AEs** | | | | | | | | | | | | | | | | | | | | |
| **AEs** | **2259**  **(9 RCTs)** | | **Serious** | **Not serious** | **Not serious** | | | **serious** | | | | **None** | | **1.36 (1.03 to 1.79)** | | | | | | ⨁⨁◯◯  **LOW** |
|  |  | |  |  |  | | |  | | | |  | |  | | | | | |  |
|  | | | | | | | | | | | | | | | | | | | | |
| *** Certainty of evidence according to Grading of Recommendations, Assessment, Development and Evaluations (GRADE):**  **High: We are very confident in the estimated effect**  **Moderate: Our confidence in the estimated effect is moderate**  **Low: We have limited confidence in the estimated effect**  **Very low: We have very little confidence in the estimated effect** | | | | | | | | | | | | | | | | | | | | |
| ***Certainty of evidence was assessed using the Grading of Recommendations Assessment, Development and Evaluation (GRADE) approach. † Effect estimates are presented as standardized mean differences (SMDs) for continuous outcomes and risk ratios (RRs) for binary outcomes. RCTs: randomized controlled trials; BPI-S: Brief Pain Inventory–Severity; BPI-I: Brief Pain Inventory–Interference; WOMAC: Western Ontario and McMaster Universities Osteoarthritis Index; VAS: Visual Analogue Scale; NRS: Numeric Rating Scale; HADS: Hospital Anxiety and Depression Scale; HAMA: Hamilton Anxiety Rating Scale; AEs: adverse events.** | | | | | | | | | | | | | | | | | | | | |
|  | | | | | | | | | | | | | | | | | | | | |

**Supplementary Table S2. Univariable meta-regression analyses of WOMAC outcomes**

| **Outcome** | **Covariate** | **β coefficient** | **95% CI** | **P value** | **R² (%)** |
| --- | --- | --- | --- | --- | --- |
| WOMAC Total | Dose | 0.0055 | −0.0216 to 0.0326 | 0.69 | 0.0 |
| WOMAC Total | Duration | 0.0071 | −0.0168 to 0.0310 | 0.56 | 0.0 |
| WOMAC Total | Study quality | −0.1574 | −0.7239 to 0.4090 | 0.59 | 0.0 |
| WOMAC Pain | Dose | −0.0015 | −0.0113 to 0.0084 | 0.77 | 0.0 |
| WOMAC Pain | Duration | −0.0007 | −0.0342 to 0.0328 | 0.97 | 0.0 |
| WOMAC Pain | Study quality | −0.1247 | −0.3138 to 0.0644 | 0.20 | 0.0 |
| WOMAC Physical Function | Dose | 0.0012 | −0.0037 to 0.0061 | 0.64 | 0.0 |
| WOMAC Physical Function | Duration | 0.0020 | −0.0101 to 0.0142 | 0.74 | 0.0 |
| WOMAC Physical Function | Study quality | −0.0541 | −0.2090 to 0.1009 | 0.49 | 0.0 |
| WOMAC Stiffness | Dose | −0.0013 | −0.0080 to 0.0055 | 0.71 | 0.0 |
| WOMAC Stiffness | Duration | −0.0002 | −0.0248 to 0.0244 | 0.99 | 0.0 |
| WOMAC Stiffness | Study quality | −0.1099 | −0.2371 to 0.0172 | 0.090 | 13.9 |

β coefficients represent the change in effect size associated with a one-unit increase in each study-level covariate. Meta-regression analyses were performed using random-effects models with restricted maximum likelihood (REML) estimation. R² represents the proportion of between-study heterogeneity explained by the covariate. CI=confidence interval; REML=restricted maximum likelihood; WOMAC=Western Ontario and McMaster Universities Osteoarthritis Index.

**Supplementary Table S3. Duration subgroup analyses of WOMAC outcomes**

| **Outcome** | **Short-term** | **Long-term** | **P** |
| --- | --- | --- | --- |
| WOMAC Total | −1.117 (−2.815, 0.581) | −0.574 (−1.571, 0.423) | 0.34 |
| WOMAC Pain | −0.463 (−2.745, 1.818) | −0.326 (−0.625, −0.028) | 0.51 |
| WOMAC Physical Function | −0.566 (−3.487, 2.354) | −0.388 (−0.597, −0.180) | 0.46 |
| WOMAC Stiffness | −0.424 (−3.123, 2.275) | −0.210 (−0.410, −0.009) | 0.34 |

**Supplementary Table S4 Study-to-analysis mapping for included randomized comparisons**

| **No.** | **Study** | **Trial arms / comparator** | **Comparator category** | **Duloxetine regimen** | **Treatment duration** | **Contributed analyses** |
| --- | --- | --- | --- | --- | --- | --- |
| 1 | Chappell 2011 | Duloxetine vs placebo | Placebo-controlled | 60–120 mg/day, once daily | 13 weeks | WOMAC, BPI-S, BPI-I, SF-36, AEs |
| 2 | Uchio 2018 | Duloxetine vs placebo | Placebo-controlled | 20 mg/day for 1 week, 40 mg/day for 1 week, then 60 mg/day | 14 weeks | WOMAC, BPI-S, SF-36, AEs |
| 3 | Sofat 2017 | Duloxetine vs placebo  duloxetine vs pregabalin | Placebo-controlled  active pharmacological comparator | 30 mg/day initially, increased to 60 mg/day, then reduced to 30 mg/day before the end of treatment | 13 weeks | AUSCAN, NRS, HADS, SAEs |
| 4 | Frakes 2011 | Duloxetine vs placebo | Placebo-controlled | 60–120 mg/day | 8–10 weeks | WOMAC, BPI-S, BPI-I, AEs |
| 5 | Chappell 2009 | Duloxetine vs placebo | Placebo-controlled | 60–120 mg/day | 13 weeks | WOMAC, BPI-S, BPI-I, SF-36, mental symptoms, AEs |
| 6 | Abou-Raya 2012 | Duloxetine vs placebo | Placebo-controlled | 60 mg/day | 16 weeks | WOMAC, mental symptoms |
| 7 | Wang G 2017 | Duloxetine vs placebo | Placebo-controlled | 30 mg/day for 1 week, then 60 mg/day | 13 weeks | WOMAC, BPI-S, BPI-I, AEs |
| 8 | Wang 2018 | Duloxetine vs usual care | Active/usual-care comparator | 60 mg/day | 6 months | VAS, treatment failure / ineffective response |
| 9 | Wang 2018 | Duloxetine + loxoprofen/glucosamine vs loxoprofen/glucosamine | Add-on / combination therapy | Duloxetine 30–60 mg/day added to loxoprofen and glucosamine | 8 weeks | WOMAC, VAS, HAMA, AEs |
| 10 | Huang 2023 | Duloxetine + radiofrequency therapy vs radiofrequency therapy | Add-on / background therapy | 30 mg on day 1, then 30 mg twice daily | 8 weeks | WOMAC, VAS, SF-36 |
| 11 | Jin 2020 | Duloxetine + surgery vs surgery | Add-on / background therapy | 40 mg/day initially, increased to 60 mg/day from week 2 | 8 weeks | VAS, treatment failure / ineffective response |
| 12 | Ma 2024 | Duloxetine vs celecoxib | Active pharmacological comparator | 60 mg in two divided doses | Not clearly reported | WOMAC, VAS |
| 13 | Yang 2024 | Duloxetine vs celecoxib plus ibuprofen | Active pharmacological comparator | Duloxetine 20 mg twice daily | 8 weeks | VAS |
| 14 | van den Driest 2022 | Duloxetine + usual care vs usual care | Add-on / background therapy | 30 mg/day for 1 week, then 60 mg/day | 12 months | WOMAC |
| 15 | Blikman 2022 | Duloxetine vs usual care | Active/usual-care comparator | 30 mg/day weeks 1–2, 60 mg/day weeks 3–8, 30 mg/day weeks 9–10 | 10 weeks | SF-36 / usual-care comparator outcomes |
| 16 | Panezai 2021 | Duloxetine + oral NSAIDs vs NSAIDs | Add-on / background therapy | 30 mg/day initially, increased to 60 mg/day after 4 weeks | 24 months | WOMAC, VAS |
| 17 | Li 2024, Pain Physician | Duloxetine + HA + CS injection vs HA + CS injection | Add-on / combination therapy | Oral duloxetine added to intra-articular HA + CS | 24 weeks | WOMAC, VAS, HAMA, AEs |
| 18 | Illeez 2022 | Duloxetine vs pregabalin | Active pharmacological comparator | 60 mg/day | 12 weeks | WOMAC, VAS, SF-36 |
| 19 | Li 2024, Contemporary Chinese Medicine | Duloxetine + NSAIDs vs NSAIDs | Add-on / background therapy | 30 mg/day initially, increased to 60 mg/day after 2 weeks | 8 weeks | WOMAC, VAS, mental symptoms, AEs |
| 20 | Hu 2023 | Duloxetine vs meloxicam | Active pharmacological comparator | 60 mg/day | 6 months | SF-36, AEs |

Comparator categories were defined according to the clinical question addressed: placebo-controlled, active pharmacological or usual-care comparator, and add-on/background therapy. Contributed analyses indicate the outcome-specific quantitative syntheses to which each study contributed; not all outcomes reported by a study necessarily contributed to a meta-analysis. In multi-arm trials, shared intervention or comparator groups were split only when more than one comparison from the same trial entered the same meta-analysis. Abbreviations: AEs, adverse events; BPI-I, Brief Pain Inventory interference; BPI-S, Brief Pain Inventory severity; CS, corticosteroid; HA, hyaluronic acid; HADS, Hospital Anxiety and Depression Scale; HAMA, Hamilton Anxiety Rating Scale; NSAIDs, non-steroidal anti-inflammatory drugs; NRS, numeric rating scale; SAEs, serious adverse events; SF-36, Short Form-36; VAS, visual analogue scale; WOMAC, Western Ontario and McMaster Universities Osteoarthritis Index.

Each included study was mapped according to comparator type and then to the outcome domains reported. When multiple outcomes or domains were reported from the same participants, each outcome was meta-analysed separately. Cross-outcome overall estimates were not calculated. For multi-arm trials, shared groups were split when more than one comparison from the same study contributed to the same meta-analysis.

Supplementary Table S5. GRADE certainty assessment for add-on/background therapy analyses

| **Outcome** | **No. of participants (studies)** | **Risk of bias** | **Inconsistency** | **Indirectness** | **Imprecision** | **Other** | **Effect estimate (95% CI)** | **Certainty** |
| --- | --- | --- | --- | --- | --- | --- | --- | --- |
| WOMAC total score | 326 (3 RCTs) | Serious | Serious | Serious | Serious | None | −0.62 (−2.34 to 1.10) | ⨁◯◯◯ Very low |
| WOMAC physical functioning | 330 (3 RCTs) | Serious | Serious | Serious | Serious | None | −0.51 (−1.19 to 0.18) | ⨁◯◯◯ Very low |
| WOMAC stiffness | 330 (3 RCTs) | Serious | Serious | Serious | Serious | None | −0.43 (−1.20 to 0.35) | ⨁◯◯◯ Very low |
| WOMAC pain | 330 (3 RCTs) | Serious | Serious | Serious | Serious | None | −0.44 (−1.19 to 0.30) | ⨁◯◯◯ Very low |
| VAS | 398 (4 RCTs) | Serious | Serious | Serious | Serious | None | −0.95 (−2.16 to 0.25) | ⨁◯◯◯ Very low |
| HAMA | 270 (2 RCTs) | Serious | Serious | Serious | Serious | None | −1.11 (−11.07 to 8.85) | ⨁◯◯◯ Very low |
| Overall AEs | 300 (2 RCTs) | Serious | Not serious | Serious | Serious | None | 1.20 (0.27 to 5.34) | ⨁◯◯◯ Very low |

AEs, adverse events; HAMA, Hamilton Anxiety Rating Scale; RCT, randomized controlled trial; VAS, visual analogue scale; WOMAC, Western Ontario and McMaster Universities Osteoarthritis Index. Effect estimates are presented as standardized mean differences for continuous outcomes and risk ratios for adverse events. Certainty was downgraded mainly because of clinical heterogeneity across background or concomitant treatments, small numbers of contributing studies and participants, imprecision, and inconsistency.

**Supplement_Merh. Search strategies**

**Pubmed**

((((((((((("Osteoarthritis"[Mesh]) OR (Osteoarthritides[Title/Abstract])) OR (Osteoarthrosis[Title/Abstract])) OR (Osteoarthroses[Title/Abstract])) OR (Arthritis, Degenerative[Title/Abstract])) OR (Arthritides, Degenerative[Title/Abstract])) OR (Degenerative Arthritides[Title/Abstract])) OR (Degenerative Arthritis[Title/Abstract])) OR (Arthrosis[Title/Abstract])) OR (Arthroses[Title/Abstract])) OR (Osteoarthrosis Deformans[Title/Abstract])) AND (((((((((((((((((("Duloxetine Hydrochloride"[Mesh]) ) OR (Hydrochloride, Duloxetine[Title/Abstract])) OR (Duloxetine HCl[Title/Abstract])) OR (HCl, Duloxetine[Title/Abstract])) OR (LY 248686[Title/Abstract])) OR (LY-248686[Title/Abstract])) OR (LY248686[Title/Abstract])) OR (Duloxetine Ethanedioate (1:1), (+-)-isomer - T353987[Title/Abstract])) OR (LY 227942[Title/Abstract])) OR (LY-227942[Title/Abstract])) OR (LY227942[Title/Abstract])) OR (Duloxetine[Title/Abstract])) OR (N-methyl-3-(1-naphthalenyloxy)-3-(2-thiophene)propanamide[Title/Abstract])) OR (N-methyl-3-(1-naphthalenyloxy)-2-thiophenepropanamine[Title/Abstract])) OR (Duloxetine, (+)-isomer[Title/Abstract])) OR (Cymbalta[Title/Abstract]))

**Embase**

#1 osteoarthritides:ti,ab,kw OR osteoarthrosis:ti,ab,kw OR osteoarthroses:ti,ab,kw OR 'arthritis, degenerative':ti,ab,kw OR 'arthritides, degenerative':ti,ab,kw OR 'degenerative arthritides':ti,ab,kw OR 'degenerative arthritis':ab,ti OR arthrosis:ti,ab,kw OR arthroses:ti,ab,kw OR 'osteoarthrosis deformans':ti,ab,kw

#2 'osteoarthritis'/exp

#3 #1 OR #2

#4 'hydrochloride, duloxetine':ti,ab,kw OR 'duloxetine hcl':ti,ab,kw OR 'hcl, duloxetine':ti,ab,kw OR 'ly 248686':ti,ab,kw OR ly248686:ti,ab,kw OR ('duloxetine ethanedioate':ab,ti AND '1:1':ab,ti AND ,:ab,ti AND +-:ab,ti AND '-isomer - t353987':ab,ti) OR 'ly 227942':ti,ab,kw OR ly227942:ti,ab,kw OR duloxetine:ti,ab,kw OR ('n methyl 3':ti,ab,kw AND '1 naphthalenyloxy':ti,ab,kw AND -3-:ti,ab,kw AND '2 thiophene':ti,ab,kw AND propanamide:ti,ab,kw) OR ('n methyl 3':ti,ab,kw AND '1 naphthalenyloxy':ti,ab,kw AND '2 thiophenepropanamine':ti,ab,kw) OR (duloxetine,:ti,ab,kw AND +:ti,ab,kw AND -isomer:ti,ab,kw) OR cymbalta:ti,ab,kw

#5 'duloxetine'/exp

#6 #4 OR #5

#7 #3 AND #6

**Web of Science**

1: Osteoarthritis (Topic) OR Osteoarthritides (Topic) OR Osteoarthrosis (Topic) OR Osteoarthroses (Topic) OR Arthritis, Degenerative (Topic) OR Arthritides, Degenerative (Topic) OR Degenerative Arthritides (Topic) OR Degenerative Arthritis (Topic) OR Arthrosis (Topic) OR Arthroses (Topic) OR Osteoarthrosis Deformans (Topic) and Preprint Citation Index (Exclude – Database)

2: Duloxetine (Topic) OR Hydrochloride, Duloxetine (Topic) OR Duloxetine HCl (Topic) OR HCl, Duloxetine (Topic) OR LY 248686 (Topic) OR LY-248686 (Topic) OR LY248686 (Topic) OR Duloxetine Ethanedioate (1:1), (+-)-isomer - T353987 (Topic) OR LY 227942 (Topic) OR LY-227942 (Topic) OR LY227942 (Topic) OR Duloxetine (Topic) OR N-methyl-3-(1-naphthalenyloxy)-3-(2-thiophene)propanamide (Topic) AND N-methyl-3-(1-naphthalenyloxy)-2-thiophenepropanamine (Topic) OR Duloxetine, (+)-isomer (Topic) OR Cymbalta (Topic) and Preprint Citation Index (Exclude – Database)

3: #2 AND #1 and Preprint Citation Index (Exclude – Database)

4: #2 AND #1 and Preprint Citation Index (Exclude – Database)

**Scopus**

( TITLE-ABS-KEY ( "Osteoarthritis" OR "Osteoarthritides" OR "Osteoarthrosis" OR "Osteoarthroses" OR "Degenerative Arthritis" OR "Degenerative Arthritides" OR "Arthrosis" OR "Arthroses" OR "Osteoarthrosis Deformans" ) ) AND ( TITLE-ABS-KEY ( "Duloxetine" OR "Duloxetine HCl" OR "Hydrochloride, Duloxetine" OR "LY 248686" OR "LY-248686" OR "Duloxetine Ethanedioate" OR "Cymbalta" ) )

**Cochrane Central Register of Controlled Trials (CENTRAL)**

#1	(Osteoarthritis):ti,ab,kw AND (Duloxetine):ti,ab,kw (Word variations have been searched)

#2	MeSH descriptor: [Osteoarthritis] explode all trees

#3	MeSH descriptor: [Duloxetine Hydrochloride] explode all trees

#4	#2 AND #3

#5	#1 OR #4

**VIP**

((((((((Title or Keywords=Knee Osteoarthritis OR Title or Keywords=Knee Osteoarthrosis) OR Title or Keywords=Osteoarthritis) OR Title or Keywords=Knee Osteoarthropathy) OR Title or Keywords=Osteoarthropathy) OR Title or Keywords=Osteoarthropathy) OR Title or Keywords=Osteoarthritis) OR Title or Keywords=Knee Osteoarthritis) AND (((Title or Keywords=Duloxetine OR Title or Keywords=Cymbalta) OR Title or Keywords=Duloxetine Hydrochloride) OR Title or Keywords=D-Duloxetine Hydrochloride))

**Wanfang**

Subject: (Knee Osteoarthritis OR Knee Osteoarthritis OR Osteoarthritis OR Knee Osteoarthropathy OR Osteoarthropathy OR Osteoarthritis OR Knee Osteoarthritis) and Subject: (Duloxetine OR Celebrex OR Duloxetine Hydrochloride)

**CNKI**

( TI=‘knee osteoarthritis’ OR TI=‘knee osteoarthrosis’ OR TI=‘osteoarthritis’ OR TI=‘knee osteoarthrosis’ OR TI=‘osteoarthrosis’ OR TI=‘osteoarthrosis’ OR TI='knee osteoarthritis' OR KY=‘knee osteoarthritis’ OR KY=‘knee osteoarthritis’ OR KY=‘osteoarthritis’ OR KY=‘knee osteoarthrosis’ OR KY=‘osteoarthrosis’ OR KY=‘osteoarthrosis’ OR KY=‘osteoarthritis’ OR KY=‘knee osteoarthritis’ ) AND ( TI=‘Duloxetine’ OR TI=‘Cymbalta’ OR TI=‘Duloxetine Hydrochloride’ OR TI=‘Dextrooxetrine Hydrochloride’ OR KY=‘Duloxetine’ OR KY=‘Cymbalta’ OR KY=‘Duloxetine Hydrochloride’ OR KY=‘Dextrooxetrine Hydrochloride’ )

**Figure S1. Supplementary efficacy and publication-bias analyses.**(A) Forest plots of Australian/Canadian Hand Osteoarthritis Index (AUSCAN) pain, stiffness, and function outcomes. (B–D) Funnel plots for Western Ontario and McMaster Universities Osteoarthritis Index (WOMAC), visual analogue scale (VAS), and adverse-event analyses, respectively.
